# Global epidemiology of type 2 diabetes in patients with NAFLD or MAFLD: a systematic review and meta-analysis

**DOI:** 10.1186/s12916-024-03315-0

**Published:** 2024-03-06

**Authors:** Limin Cao, Yu An, Huiyuan Liu, Jinguo Jiang, Wenqi Liu, Yuhan Zhou, Mengyuan Shi, Wei Dai, Yanling Lv, Yuhong Zhao, Yanhui Lu, Liangkai Chen, Yang Xia

**Affiliations:** 1 The Third Central Hospital of Tianjin, Tianjin, China; 2 Department of Endocrinology, Beijing Chao-Yang Hospital, Capital Medical University, Beijing, China; 3 Department of Clinical Epidemiology, Shengjing Hospital of China Medical University, No. 36, San Hao Street, Shenyang, Liaoning, 110004 China; 4 Liaoning Key Laboratory of Precision Medical Research On Major Chronic Disease, Liaoning Province, Shenyang, China; 5 Department of Nutrition and Food Hygiene, Hubei Key Laboratory of Food Nutrition and Safety, School of Public Health, Tongji Medical College, Huazhong University of Science and Technology, Wuhan, 430030 China; 6 School of Nursing, Peking University, 38 Xueyuan Rd, Haidian District, Beijing, 100191 China

**Keywords:** Prevalence, Incidence density, Non-alcoholic fatty liver disease, Metabolic-associated fatty liver disease, Type 2 diabetes

## Abstract

**Background:**

Non-alcoholic fatty liver disease (NAFLD) and metabolic-associated fatty liver disease (MAFLD) shares common pathophysiological mechanisms with type 2 diabetes, making them significant risk factors for type 2 diabetes. The present study aimed to assess the epidemiological feature of type 2 diabetes in patients with NAFLD or MAFLD at global levels.

**Methods:**

Published studies were searched for terms that included type 2 diabetes, and NAFLD or MAFLD using PubMed, EMBASE, MEDLINE, and Web of Science databases from their inception to December 2022. The pooled global and regional prevalence and incidence density of type 2 diabetes in patients with NAFLD or MAFLD were evaluated using random-effects meta-analysis. Potential sources of heterogeneity were investigated using stratified meta-analysis and meta-regression.

**Results:**

A total of 395 studies (6,878,568 participants with NAFLD; 1,172,637 participants with MAFLD) from 40 countries or areas were included in the meta-analysis. The pooled prevalence of type 2 diabetes among NAFLD or MAFLD patients was 28.3% (95% confidence interval 25.2–31.6%) and 26.2% (23.9–28.6%) globally. The incidence density of type 2 diabetes in NAFLD or MAFLD patients was 24.6 per 1000-person year (20.7 to 29.2) and 26.9 per 1000-person year (7.3 to 44.4), respectively.

**Conclusions:**

The present study describes the global prevalence and incidence of type 2 diabetes in patients with NAFLD or MAFLD. The study findings serve as a valuable resource to assess the global clinical and economic impact of type 2 diabetes in patients with NAFLD or MAFLD.

**Supplementary Information:**

The online version contains supplementary material available at 10.1186/s12916-024-03315-0.

## Background

Type 2 diabetes is a worldwide public health burden, which accounts for about 90–95% of diabetes mellitus [1]. Over the past 40 years, the number of adults with diabetes mellitus has quadrupled from 108 million in 1980 to 463 million in 2019 globally [2, 3]. This figure is predicted to be 579 million in 2030 and 700 million in 2045 [2, 4]. Moreover, it is estimated that the amount of direct health care expenditures attributed to type 2 diabetes in 2007 was $232 billion, whereas in 2019, it soared to $760 billion [5]. Thus, it is essential to curb the rise of type 2 diabetes in order to minimize economic expenses and protect individuals and communities from its enduring negative consequences.

Non-alcoholic fatty liver disease (NAFLD) is the most common cause of chronic liver disease worldwide, encompassing a spectrum that includes hepatic steatosis, non-alcoholic steatohepatitis (NASH), advanced liver fibrosis, cirrhosis, and hepatocellular carcinoma (HCC). Currently, one in four people in the world suffers from NAFLD [6]. Eventually, 20 million people will die from NAFLD-related liver disease [7]. In clinical settings, NAFLD has been reported to share similar pathophysiological mechanism (such as insulin resistance, defective hepatic lipidic profile, and abnormal triglyceride metabolism) with type 2 diabetes [8–10] and is strongly associated with it [9, 11, 12]. A recent meta-analysis has revealed that NAFLD increases the risk of type 2 diabetes incident by approximately twofold [13]. Therefore, reducing the prevalence of NAFLD could help mitigate the impact of type 2 diabetes to some extent. However, to the best of our knowledge, there are no reports on global prevalence and incidence density of type 2 diabetes among NAFLD patients that can be used to delineate its epidemiological features [13–17].

Furthermore, given that NAFLD is closely related to several metabolic diseases, the term metabolic-associated fatty liver disease (MAFLD) was proposed in 2020 to better describe these coexisting conditions and reflect the etiology of fatty liver diseases [18, 19]. MAFLD is defined based on evidence of hepatic steatosis and is simultaneously accompanied by at least one of the following conditions: obesity, diabetes, or metabolic dysregulation [18]. Of note, these conditions are closely related to type 2 diabetes. However, no research to date has reported on the epidemiological features of type 2 diabetes among MAFLD patients.

Therefore, the present study utilized a comprehensive meta-analysis to report the worldwide incidence and prevalence of type 2 diabetes among individuals with NAFLD or MAFLD.

## Methods

### Search terms, included and excluded criteria

The present systematic review and meta-analysis were in accordance with the Preferred Reporting Items for Systematic Reviews and Meta-Analyses reporting guidelines [20]. The study protocol was registered in PROSPERO with an ID number of CRD42022335692 (https://www.crd.york.ac.uk/prospero/).

Two researchers independently conducted a literature search in the PubMed, EMBASE, MEDLINE, and Web of Science databases in the period from their inception to December 2022 using a combination of the following search terms related to type 2 diabetes in NAFLD or MAFLD: "fatty liver" (OR "NAFLD" OR "nonalcoholic fatty liver disease" OR "nonalcoholic steatohepatitis "OR "hepatic steatosis" OR "MAFLD" OR " metabolic associated fatty liver disease") AND "diabetes" NOT "animals"[mesh]. The search included all observational studies conducted in adults (≥ 18 years old) published in peer-reviewed journals until December 2022. In addition to the initial database search, references obtained from the retrieved publications were also searched to identify any additional articles that may have been missed.

The following publications were excluded: reviews or abstracts; studies that were unable to ascertain the NAFLD diagnosis; studies on patients with viral hepatitis B and C (HBV/HCV), or other liver-related disease or excess alcohol consumption; studies with patients under 18 years old or pregnant individuals; studies that did not report screening for excess alcohol use; studies reporting on type 1 diabetes; studies that included only groups with certain metabolic condition, such as morbidly obese patients; and studies not published in the English language. If multiple studies were conducted using the same population, the most recent data were utilized for the present analysis.

### Data extraction and analysis

Usable data from eligible studies were independently extracted by two authors (H. L. and J. J.). Any discrepancies in their decisions were resolved through consultation with a third author (L. C.). The following data were extracted from each study: title, author (s), study year, publication year, study location (country and region), study design, NAFLD diagnosis, sample size, average age, percentage of males, and number of participants with NAFLD or MAFLD.

To estimate the pooled prevalence, the prevalence rates were combined in random-effects meta-analyses (normal-normal model) that took into account inter-study heterogeneity. Moreover, meta-analysis was performed using logit transformed proportions to obtain better statistical properties [21]. To address the potential for misestimation due to liver index and hospital records of NAFLD definition, a sensitivity analysis was performed after excluding those studies, based on previous reports [5, 6]. The percentage of males, average age of the sample population, geographic region, diagnostic method, follow-up duration, and publication year were examined both in univariate and multivariate meta-regression models. A funnel plot, Begg-Mazumdar’s rank correlation test, and Egger’s regression test were used to estimate whether there were any publication or related biases. The detailed data analysis was observed in Additional file 1. Supplementary Methods [5, 6, 21–23].

In addition, Joinpoint Regression Analysis was used to calculate the average annual percent change (AAPC) and the corresponding 95% CIs in the temporal trend of each part with natural log-transformed rates. All analyses were performed using the Stata version 17.0 software (Stata Corp, College Station, TX, USA), R version 4.0.5, and Joinpoint version 4.7.0.0. Bilateral *P* < 0.05 was considered as statistically significant.

Furthermore, GRADEpro version 3.6.1 was used to assess evidence quality and grading of recommendation strength in the included studies in the quantitative synthesis—meta-analysis, in accordance to the grading of recommendation, assessment, and evaluation (GRADE) instrument [24]. This assessment was based on the considerations of study design, consistency, directness, heterogeneity, precision, publication bias, and other aspects reported by studies included in this systematic review. The quality of the evidence was rated as high, moderate, low, or very low [25].

### Patient and public involvement statement

Patients and/or the public were not involved in the design, conduct, reporting or dissemination plans of this research.

## Results

The electronic search yielded 26,131 articles (Additional file 2: Fig. S1). A total of 20,017 records were retained after removing the duplicates. Furthermore, 18,116 records with ineligible titles and abstracts were excluded based on the aforementioned exclusion criteria, in addition to 1506 records with no available information, not describing observational studies, including teenage subjects, not written in English, without baseline data, and with the same study participants. As a result, 395 reports were included in the final study analysis. Of those, 364 studies focused on the epidemiological research on type 2 diabetes in NAFLD patients, while 42 studies reported on the MAFLD research.

### Prevalence of type 2 diabetes among patients with NAFLD or MAFLD

**Fig. 1:**
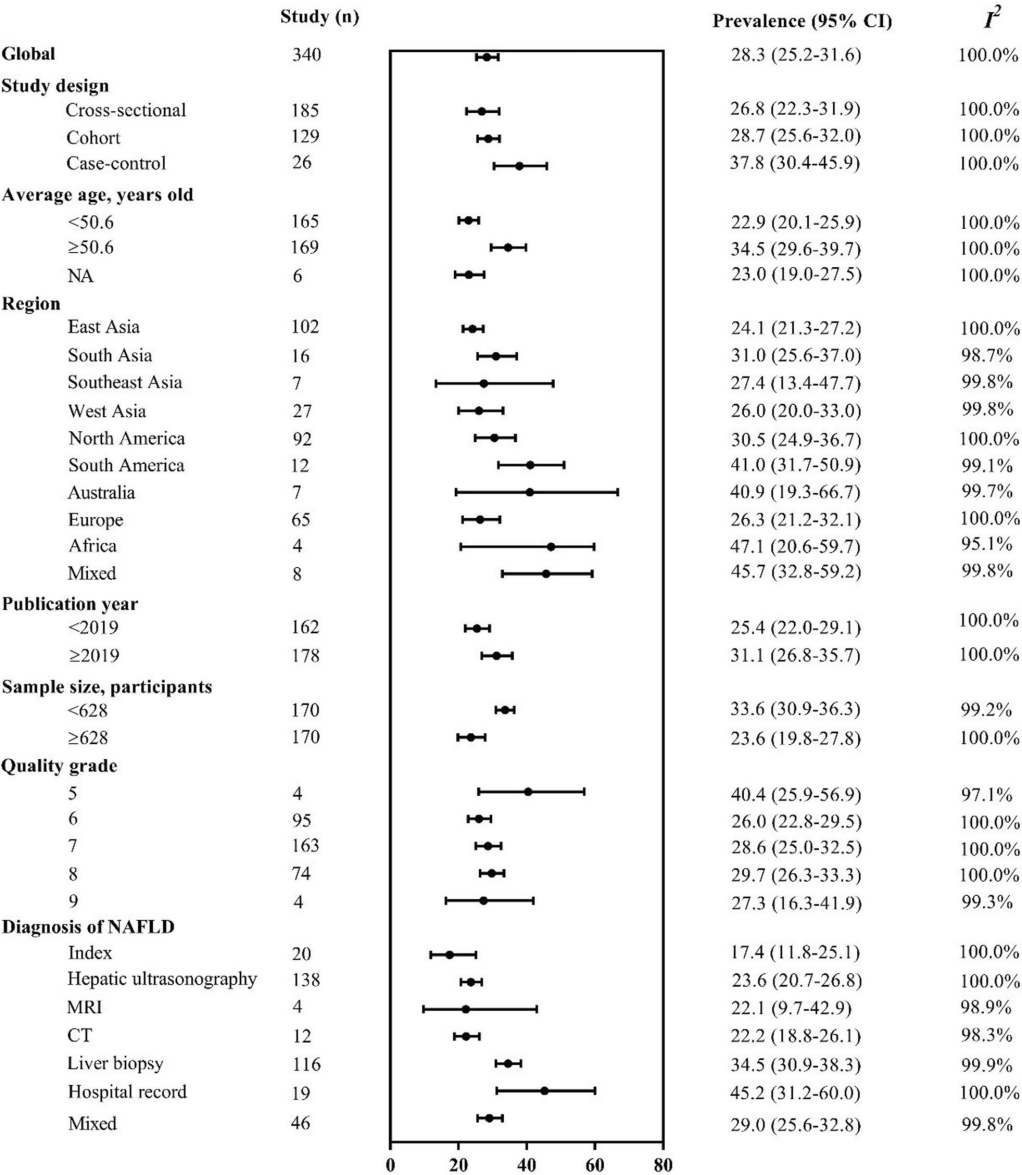
The prevalence of type 2 diabetes among patients with NAFLD—stratified by age, region, publication year, sample size, and diagnosis of NAFLD. Mixed diagnostic methods refer to the definition of NAFLD in a study using more than one diagnostic method

**Fig. 2:**
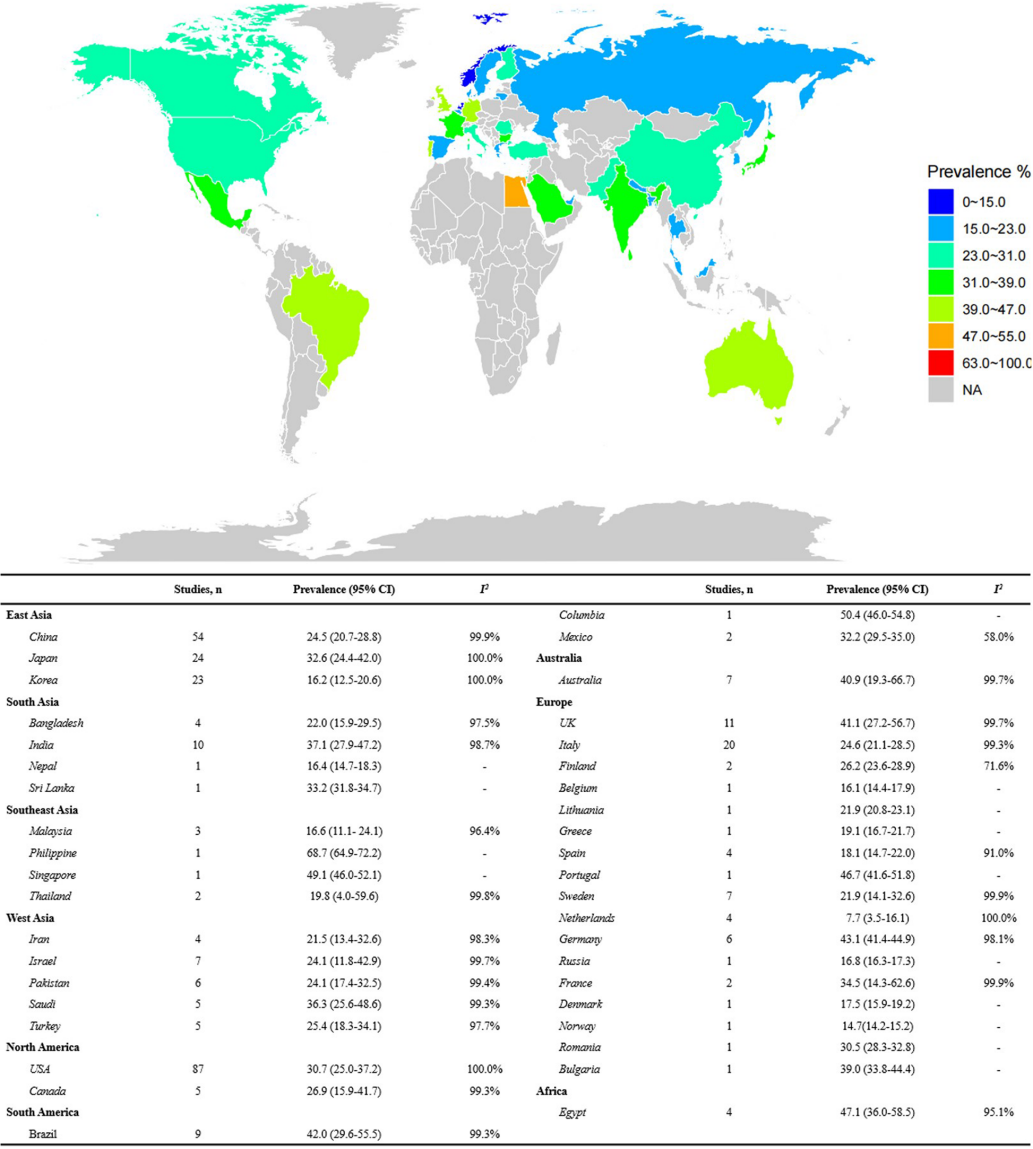
The prevalence of type 2 diabetes among patients with NAFLD across 40 countries

The pooled global prevalence of type 2 diabetes among patients with NAFLD or MAFLD was 28.1% (95% confidence interval [CI] 25.1–31.3%). Specifically, a total of 340 studies including a total 5,563,639 patients with NAFLD were included in the study (Additional file 3: Table S1 [26–362]). Briefly, the median age of these participants was 50.6 (range 33.1–78.0) years, with 51.2% of NAFLD patients being male (range 9.0–94.3%) (Additional file 3: Table S2). The estimated global prevalence of type 2 diabetes among patients with NAFLD was 28.3% (95% confidence interval [CI] 25.2–31.6%; Fig. 1). The prevalence of type 2 diabetes among patients with NAFLD was dramatically increased from 28.4% (95% CI 24.4–32.7%) in 2002 to 34.1% (95% CI 30.8–37.5%) in 2022 (Additional file 2: Fig. S2), similar to the results of the AAPC, especially for Australia, South America, and Southeast Asia (all *P* < 0.05) (Additional file 3: Table S3). Across the region, pooled prevalence estimates ranged from 24.1% in East Asia (*n* = 102; 95% CI 21.3–27.2%; *I*^*2*^ = 100.0%) to 47.1% in Africa (*n* = 4; 95% CI 20.6–59.7%; *I*^*2*^ = 95.1%). The pooled prevalence of type 2 diabetes in NAFLD patients across countries is listed in Fig. 2. Across diagnosis of NAFLD, the pooled prevalence of type 2 diabetes among patients with NAFLD was the lowest in index (*n* = 20; 17.4%; 95% CI 11.8–25.1%; *I*^*2*^ = 100.0%) and the highest in hospital records (*n* = 19; 45.2%; 95% CI 31.2–60.0%; *I*^*2*^ = 100.0%). Furthermore, sensitivity analyses results that excluded studies that using liver index and hospital records as NAFLD definitions, were consistent with the overall study findings (28.2% vs. 28.3%; Additional file 3: Table S4).

For secondary analysis (Additional file 3: Table S5), the pooled prevalence values for type 2 diabetes among patients with NAFLD were 15.7% (*n* = 19; 95% CI 11.7–20.7%; *I*^*2*^ = 99.8%) and 22.2% (*n* = 25; 95% CI 17.2–28.0%; *I*^*2*^ = 99.8%) for males and females, respectively. The pooled prevalence of type 2 diabetes among patients with lean-NAFLD was 15.2% (*n* = 19; 95% CI 10.8–21.0%; *I*^*2*^ = 99.5%), while the prevalence among non-lean NAFLD patients was 25.9% (*n* = 23; 95% CI 20.3–32.5%; *I*^*2*^ = 99.8%). Moreover, the present study further investigated the prevalence of type 2 diabetes among NAFLD patients with or without Corona Virus Disease 2019 (Covid-19). As shown, the prevalence of type 2 diabetes for those with Covid-19 was 18.7% (*n* = 2; 95% CI 5.8–46.1%; *I*^*2*^ = 99.2%) and 28.4% (*n* = 338; 95% CI 25.2–31.7%; *I*^*2*^ = 100.0%) for those without Covid-19.

The univariate meta-regression analysis indicated that study design (*P* = 0.02), mean age (*P* < 0.0001), proportion of males (*P* = 0.03), publication year (*P* = 0.01), and NAFLD diagnosis (*P* < 0.0001) were significantly associated with the prevalence of type 2 diabetes (Additional file 3: Table S6). In the multivariate analysis, mean age (*P* < 0.0001), publication year (*P* = 0.03), and NAFLD diagnosis (*P* < 0.0001) remained significantly associated with the prevalence of type 2 diabetes.

In addition, a total of 40 studies, including 1,169,899 patients with MAFLD were evaluated in the analyses (Additional file 3: Table S7 [171, 248, 251, 271, 307–309, 313, 335, 349, 363–391]). The median age of the participants was 50.9 (range 35.1–68.4) years, with 53.3% of MAFLD patients being male (range 23.9–85.4%; Additional file 3: Table S8). The pooled global prevalence of type 2 diabetes among patients with MAFLD was 26.2% (95% CI 23.9–28.6%; Additional file 2: Fig. S3). Across the geographic location, the highest pooled prevalence of type 2 diabetes in MAFLD patients was reported in West Asia (*n* = 1; 81.5%; 95% CI 79.3–83.5%) and the lowest prevalence in East Asia (*n* = 21; 21.1%; 95% CI 18.6–23.8%; *I*^*2*^ = 99.3%). The pooled prevalence of type 2 diabetes in MAFLD patients across countries is shown in Additional file 2: Fig. S4. Moreover, the results from AAPC analysis indicated that there was an increased trend of prevalence in type 2 diabetes populations with MAFLD (Additional file 3: Table S9). For meta-regression analysis, only geographic region (*P* < 0.05) was significantly associated with the prevalence rates both in univariate and multivariate meta-regression analyses (Additional file 3: Table S10).

### Incidence density of type 2 diabetes among patients with NAFLD or MAFLD

**Fig. 3:**
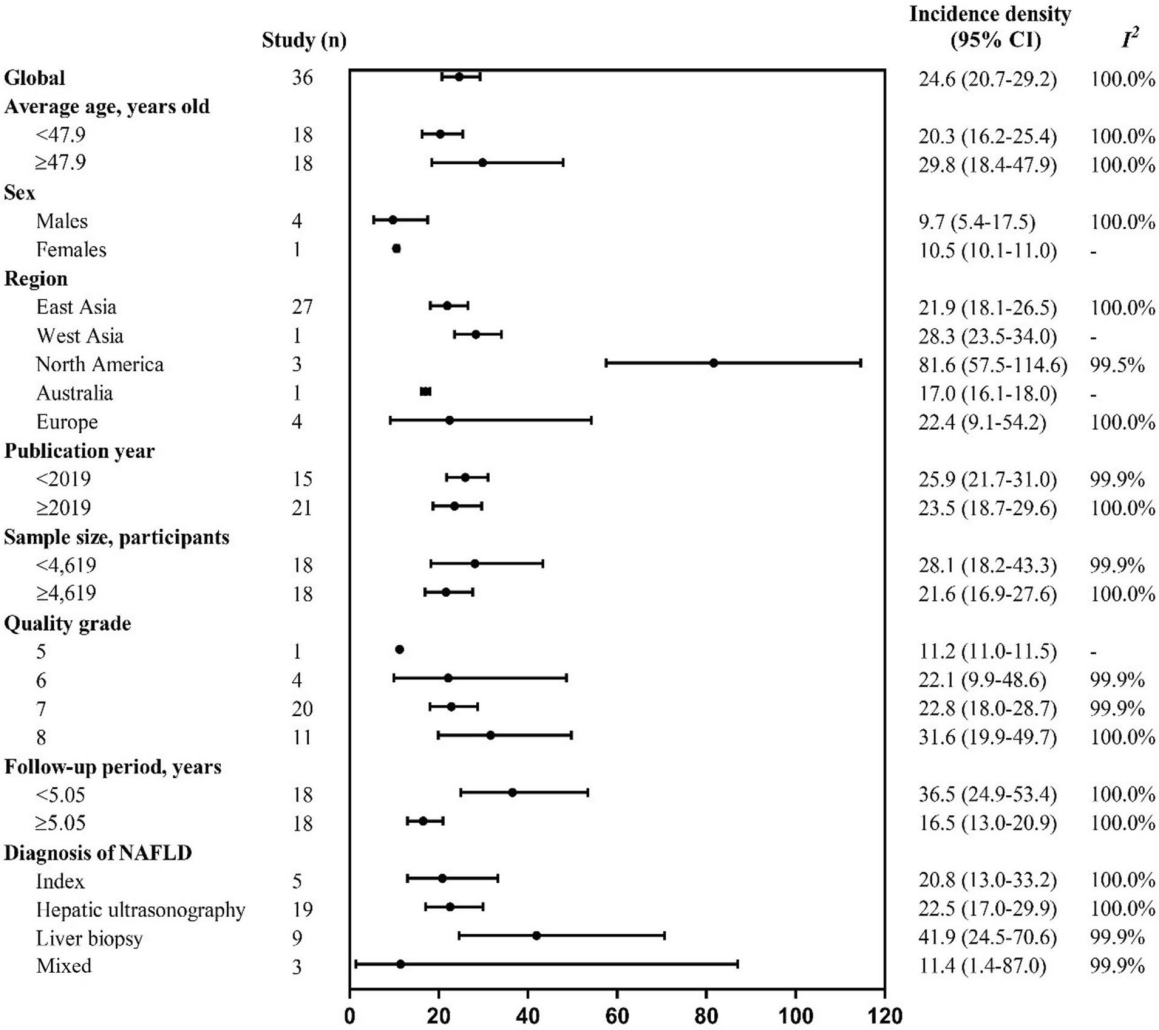
The incidence density of type 2 diabetes among patients with NAFLD—stratified by age, region, publication year, sample size, and diagnosis of NAFLD. Mixed diagnostic methods refer to the definition of NAFLD in a study using more than one diagnostic method

**Fig. 4:**
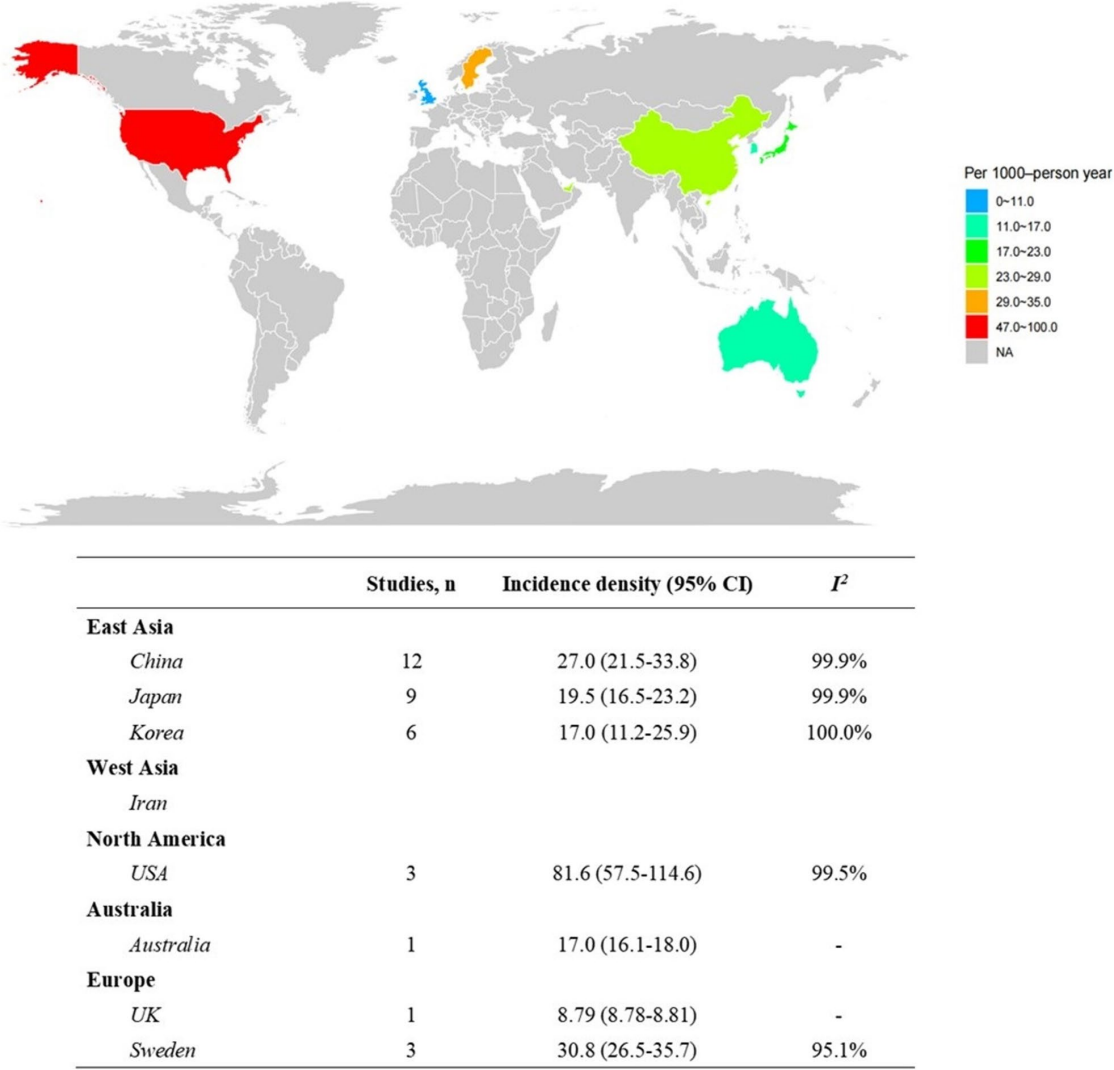
The incidence density of type 2 diabetes among patients with NAFLD across 8 countries

The pooled global incidence density of type 2 diabetes among patients with NAFLD or MAFLD was 24.0 per 1000-person year (95% CI 20.3–28.4). To be specific, a total of 36 studies evaluated in the incidence analysis, included 1,327,087 patients with NAFLD (Additional file 3: Table S11 [43, 82, 174, 203, 221, 233, 262, 274, 285, 349, 392–417]). Briefly, the median age of these participants was 48.3 (range 30.7–69.4) years, and a median follow-up time of 5.05 years. In addition, 61.2% (range 36.0–90.5) of patients with NAFLD were male (Additional file 3: Table S12). The estimated global incidence density of type 2 diabetes among patients with NAFLD was 24.6 per 1000-person year (95% CI 20.7 to 29.2; Fig. 3). There was a substantial decrease in the incidence of type 2 diabetes among patients with NAFLD from 28.3 per 1000-person year in 2003 to 16.1 per 1000-person year in 2022 (Additional file 2: Fig. S5), while no significant trend of AAPC analysis was observed (Additional file 3: Table S13). Across the region, pooled incidence density estimates ranged from 17.0 per 1000-person year (*n* = 1; 95% CI 16.1 to 18.0) in Australia to 81.6 per 1000-person year (*n* = 3; 95% CI 57.5 to 114.6; *I*^*2*^ = 99.5%) in North America. The pooled incidence density of type 2 diabetes in NAFLD patients across countries is listed in Fig. 4. Subgroup differences were significant in NAFLD diagnosis, where the pooled incidence density was the highest in liver biopsy (*n* = 9; 41.9 per 1000-person year; 95% CI 24.5 to 70.6; *I*^*2*^ = 99.9%) and the lowest in liver index (*n* = 5; 20.8 per 1000-person year; 95% CI 13.0 to 33.2; *I*^*2*^ = 100.0%), except for mixed diagnostic methods (the definition of NAFLD in a study using more than one diagnostic method). In the secondary analysis, the pooled type 2 diabetes incidence density among patients with NAFLD for males was slightly lower than that of females (9.7 vs. 10.5). Additionally, sensitivity analyses results, which excluded studies that used index and hospital records for NAFLD diagnosis, also demonstrated results similar to those observed in the whole population (Additional file 3: Table S14). Only the region (*P* = 0.04) demonstrated a significant association with the incidence density of type 2 diabetes in the univariate and multivariate analysis (Additional file 3: Table S15).

Moreover, only three studies from East Asia with a total of 5222 patients with MAFLD were included in the incidence analysis (Additional file 3: Table S16 [349, 418, 419]). The median age of these participants was 45.2 (range 40.0–54.9) years, with a median follow-up time of 10 years (Additional file 3: Table S17). Studies on the incidence density of type 2 diabetes among patients with MAFLD were all from China and had a pooled incidence density of 26.9 per 1000-person year (95% CI 7.3 to 44.4; Additional file 3: Table S18).

### Level of evidence

Overall, the quality of the evidence for the results of the GRADE system assessment was rated as very low (Additional file 2: Fig. S6). Heterogeneity was the main factor leading to the limited quality of the evidence.

## Discussion

The present study determined that the global prevalence of type 2 diabetes among NAFLD patients is 28.3%, with the lowest prevalence rate observed in East Asia. The global incidence density of type 2 diabetes among NAFLD is 24.6 per 1000-person year. The epidemiological features of type 2 diabetes among patients with MAFLD were consistent, showing a pooled prevalence of 26.2% and an incidence density of 26.9 per 1000-person year.

According to the World Health Organization (WHO), the prevalence of diabetes rose to approximately 10% (537 million) in 2019 and is expected to reach 783 million by 2045 [420]. Thus, the prevalence of type 2 diabetes is more than double that of the general population. Consistently, a previous meta-analysis has reported a global prevalence of type 2 diabetes associated with NAFLD of 22.5% for the period of 1989–2015 [6], which was lower than the values in the present study (28.3%). Although several studies have demonstrated that NAFLD is associated with a nearly two-fold increased risk of type 2 diabetes incident, compared to that in non-NAFLD individuals [15, 16], the global incidence density of type 2 diabetes in NAFLD patients remains unclear. A meta-analysis of 33 studies from 2000 to June 2020 has shown a crude incidence of 18.1% among NAFLD patients with type 2 diabetes [17]. This figure was somewhat higher than the value reported in the present findings (14.9%), which might be attributed to the untransformed rate calculation.

Based on the present meta-analysis, the epidemiological features of type 2 diabetes in NAFLD patients are highly heterogenous across geographic regions. Higher prevalence of type 2 diabetes in NAFLD patients was observed in Africa (especially North Africa), South America, and Australia, while lower rates were found in Europe and Asia. As well, results from AAPC indicated that the increased trends of type 2 diabetes among NAFLD were significant in Australia, South America, and Southeast Asia. Consistently with our findings, South America has been reported to have the highest prevalence of NAFLD in 2019, while Europe and Asia showed lower rates [421]. The highest prevalence of age-standardized diabetes worldwide was observed in the Middle East and North Africa (12.2%), and the lowest prevalence of diabetes was found in Europe (6.3%) [422]. Furthermore, higher incidence density of type 2 diabetes among NAFLD populations was noted in North America, while lower incidence was observed in Australia. Regarding to the AAPC, an increased trend of incidence density has been found in East Asia, whereas a downward trend was observed in Europe. With the rapid social and economic development of middle socio-demographic index countries and developing countries in East Asia such as China, great changes have taken place in diet structure and lifestyles. For instance, smoking has been reported to increase the prevalence of NAFLD and advanced liver fibrosis [423]. Soft drinks, the leading source of added sugar worldwide, have been linked to obesity, insulin resistance, and NAFLD [424]. However, basic infrastructure is insufficient to support a healthy lifestyle, and current health services are unable to identify the onset of diabetes in people with NAFLD early and intervene in a timely manner. In addition, population aging accelerates the occurrence of these chronic diseases. According to the WHO, there were 1 billion people worldwide over the age of 60 years in 2019, and this number will rise to 1.4 billion by 2030 and 2.1 billion by 2050 [425]. Both NAFLD and type 2 diabetes are closely associated with aging. The worldwide prevalence of NAFLD has increased from 25.5% in or before 2005 to 37.8% in 2016 or later [426]. Additionally, 135.6 million people aged 65–99 years were living with diabetes in 2019. It is estimated that the number of people over the age of 65 years with diabetes will reach 195.2 million by 2030 and 276.2 million by 2045 [427]. Thus, the increasing disease burden of type 2 diabetes among NAFLD patients is closely related to aging. Intensive measures should be planned and implemented in less developed countries to prevent further increases in the burden of disease and to strengthen health services.

The prevalence and incidence of type 2 diabetes among NAFLD patients also vary depending on the diagnostic methods for NAFLD. Although liver biopsy remains the gold standard for diagnosis of fatty liver, it has limitations due to sampling variability, invasiveness, and high cost [428]. Numerous non-invasive biomarkers, including liver index (serum markers) and imaging modalities, are preferred for practical applications. Consistent with published literature [6], the present study found that liver index can underestimate the prevalence rate of type 2 diabetes, while hospital records may overestimate it. Studies have shown that analyses using liver enzymes (blood tests only) to estimate the prevalence of NAFLD consistently yield lower results than those using imaging studies. In North America, for example, the prevalence of NAFLD diagnosed via ultrasound is 24%, but only 13% if a blood test is used [6]. Additionally, aminotransferase levels may be only mildly elevated in NAFLD individuals, so that up to 78% of patients with NAFLD may actually have normal liver enzyme levels [429]. These findings can explain why publication bias was no longer present when studies using blood tests as the sole means of diagnosing NAFLD were removed in the sensitivity analysis. However, after excluding studies using liver index or hospital records for NAFLD diagnosis, the sensitivity analyses results were still consistent with those of the main analyses, which may be attributed to the lack of studies on liver index and hospital records for NAFLD diagnosis. Clearly, these data also support the importance of selecting the most accurate method to diagnose NAFLD, such as imaging or histology [430].

Results from secondary analysis indicated a significantly higher prevalence of type 2 diabetes in non-lean NAFLD patients, compared to lean NAFLD patients. These findings were consistent with observations in the general population. As reported by a previous systematic review and meta-analysis, a similar phenomenon has been observed in the general population. A study has reported that the overall prevalence of lean NAFLD was 19.2% among the NAFLD population and 5.1% in the general population [431]. Except for the fact that obesity is a relevant risk factor for type 2 diabetes, the observed difference in the prevalence of type 2 diabetes between lean and non-lean NAFLD patients may be related to the differences in genetic variations, as well as in gut microbiota among different ethnic populations. Compared to non-lean NAFLD individuals, lean NAFLD individuals showed better metabolic and histological characteristics. In addition, a previous microbiome study has also indicated a significant difference in fecal and blood microbiota profiles between lean and non-lean NAFLD patients [432]. Additionally, the rate of type 2 diabetes in NAFLD populations has also suggested a sex difference, with the condition being more common in females than males. However, it is worth noting that the sex differences in the prevalence of type 2 diabetes and NAFLD are often thought to depend on the patients’ reproductive stage of life [433, 434]. Specifically, there are more cases of type 2 diabetes or NAFLD in males before the age of puberty, whereas postmenopausal females have a higher prevalence of type 2 diabetes or NAFLD. Studies on the sex subgroup populations are needed to better understand these differences. In addition, published evidence has reported that there were several direct links (e.g., insulin resistance, immune injury) between Covid-19 and the metabolic and endocrine systems. Not only are patients with metabolic dysfunction, such as NAFLD and diabetes, at an increased risk of developing severe Covid-19 but also infection with SARS-CoV-2 might lead to new-onset diabetes or aggravation of pre-existing metabolic disorders [10, 435]. Moreover, only two studies included adults Covid-19 patients from China and Mexico have reported that a much higher prevalence of NAFLD was observed in Covid patients than that reported in the general population (25%). Of which, these patients also had significantly higher proportions of diabetes than the non-NAFLD cases [302, 353]. On the contrary, research from the UK Biobank has revealed no causal relationship between NAFLD and Covid-19 using Mendelian Randomization Analysis [436]. Since the study samples we included were not comprehensive and representative enough, we found the pooled prevalence of type 2 diabetes in NAFLD individuals was lower among Covid-19 patients than in non-Covid-19 patients. Further studies were still warranted to explore the impact of Covid-19 on metabolic disorders.

According to the present results, the prevalence of type 2 diabetes in MAFLD patients was lower than that in NAFLD patients, with the highest prevalence in Europe and the lowest prevalence in Asia. In fact, observational data meta-analysis has reported that Europe has the highest pooled prevalence of MAFLD, followed by Asia and North America [437]. It is worth noting that there are slight differences in prevalence between NAFLD and MAFLD in published epidemiological studies, where the prevalence for MAFLD is often higher than that for NAFLD [437]. Evidence from the 2017–2018 National Health and Nutrition Examination Survey, which is a cohort study conducted in a representative sample of the general US population, has shown that the prevalence values for NAFLD and MAFLD based on imaging diagnose were 37.1 and 39.1%, respectively [438]. Since there is limited evidence regarding the prevalence of type 2 diabetes in MAFLD, further studies are still needed. Moreover, research related to the incidence density of type 2 diabetes among MAFLD patients has only been published in China. Thus, the incidence results are not directly comparable to the global values. Given the fact that the MAFLD criteria are more practical and have higher ability for identifying at-high-risk patients than that of the NAFLD criteria [251], more studies from diverse geographic locations are necessary to obtain a more comprehensive understanding of the incidence of type 2 diabetes in MAFLD patients worldwide.

A key strength of the present analysis is that it first presents a comprehensive overview of the global epidemiological features of type 2 diabetes among NAFLD and MAFLD patients. This work provides essential data to develop policies to address this rising health concern. However, several limitations warrant consideration. First, significant heterogeneity was present in the included studies, which contribute to a low evaluation of evidence quality by GRADE system. However, the quality of initial evidence of observational studies is low. Unexplored factors, such as comorbid conditions of NAFLD or MAFLD, may have contributed to the heterogeneity. Second, some variations arising from different diagnostic methodologies should be acknowledged. The use of liver enzymes to estimate the prevalence of NAFLD may underestimate the true prevalence of NAFLD, compared to liver biopsy and imaging modalities [6]. Another limitation is the scarcity of data on the incidence of type 2 diabetes among MAFLD patients from numerous countries, preventing a complete global overview. Moreover, given that we mainly focused on studies about NAFLD, those with alcohol drinking were excluded. This could underestimate the epidemiological rate of MAFLD. In addition, studies we included in the present study might not represent the geographical areas to which they belong, further national and representative studies are still warranted.

## Conclusions

The present meta-analysis results are important for primary care physicians, specialists, and health policy makers, given the increasing burden of type 2 diabetes among patients with NAFLD and/or MAFLD over the last few years. With more than one-fifth of NAFLD or MAFLD adults affected by type 2 diabetes globally, it is crucial to focus on the development and complications of NAFLD and implement strategies to mitigate its impact.

### Supplementary Information

**Additional file 1. **Supplementary methods.

**Additional file 2: Figures S1-S6. Fig. S1. **Flow chart. **Fig. S2. **The prevalence of type 2 diabetes among patients with NAFLD-stratified by publication year.** Fig. S3. **The prevalence of type 2 diabetes among patients with MAFLD across 12 countries.** Fig. S4. **The prevalence of type 2 diabetes among patients with MAFLD-stratified by age, region, publication year, sample size and quality grade.** Fig. S5. **The incidence density of type 2 diabetes among patients with NAFLD-stratified by publication year. **Fig. S6. **Grading of Recommendation, Assessment, Development, and Evaluation (GRADE) instrument.

**Additional file 3: Tables S1-S18. Table S1. **Characteristics of the included studies in the prevalence of type 2 diabetes among NAFLD patients.** Table S2. **Characteristics of studies reporting the prevalence of type 2 diabetes in patients with NAFLD: source of heterogeneity. **Table S3. **Average annual percent change (%) in the prevalence of type 2 diabetes among NAFLD populations globally and by region.** Table S4. **The prevalence of type 2 diabetes among patients with NAFLD-stratified by age, region, publication year, sample size and diagnosis of NAFLD (excluding index and hospital record of NAFLD). **Table S5. **Secondary analysis of the prevalence of type 2 diabetes among patients with NAFLD. **Table S6. **Univariable and multivariable meta-regression analyses on the prevalence of type 2 diabetes among patients with NAFLD. **Table S7. **Characteristics of the included studies in the prevalence of type 2 diabetes among MAFLD patients. **Table S8. **Characteristics of studies reporting the prevalence of type 2 diabetes in patients with MAFLD: source of heterogeneity. **Table S9. **Average annual percent change (%) in the prevalence of type 2 diabetes among MAFLD populations globally and by region. **Table S10. **Univariable and multivariable meta-regression analyses on the prevalence of type 2 diabetes among patients with MAFLD. **Table S11. **Characteristics of the included studies in the incidence density of type 2 diabetes among NAFLD patients.** Table S12. **Characteristics of studies reporting the incidence density of type 2 diabetes in patients with NAFLD: source of heterogeneity. **Table S13. **The incidence density of type 2 diabetes among patients with NAFLD-stratified by age, region, publication year, sample size and diagnosis of NAFLD (excluding index and hospital record of NAFLD). **Table S14. **Average annual percent change (%) in the incidence density of type 2 diabetes among NAFLD populations globally and by region. **Table S15. **Univariable and multivariable meta-regression analyses on the incidence density of type 2 diabetes among patients with NAFLD. **Table S16. **Characteristics of the include studies in the incidence density of type 2 diabetes among MAFLD patients. **Table S17. **Characteristics of studies reporting the incidence density of type 2 diabetes in patients with MAFLD: source of heterogeneity. **Table S18. **The incidence density of type 2 diabetes among patients with MAFLD.

## Data Availability

The study-specific summary data included in the meta-analysis can be obtained from the corresponding authors at yxia@cmu.edu.cn.

## Abbreviations

AAPC: Average annual percent change
CI: Confidence interval
Covid-19: Corona Virus Disease 2019
HCC: Hepatocellular carcinoma
MAFLD: Metabolic-associated fatty liver disease
NAFLD: Non-alcoholic fatty liver disease
NASH: Non-alcoholic steatohepatitis
WHO: World Health Organization
