# Supplementary material for: Global epidemiology of type 2 diabetes in patients with NAFLD or MAFLD: a systematic review and meta-analysis
Source: BMC Med. 2024 Mar 6;22:101. doi: 10.1186/s12916-024-03315-0 (PMC10919055; doi:10.1186/s12916-024-03315-0)
Supplement: Supplementary file 1 — Additional file 1. Supplementary methods. [file 12916_2024_3315_MOESM1_ESM.docx]

**Additional File 1: Supplementary Methods.**

NAFLD was diagnosed using one of the following methods: hospital records, ultrasound examination, CT scanning, MRI, index (e.g., hepatic steatosis index, fatty liver index), or liver biopsy in the absence of excessive alcohol consumption and other liver-related diseases (e.g., viral hepatitis, auto-immune hepatitis, haemochromatosis, and Wilson’s disease).

The Newcastle-Ottawa scale (NOS) score (ranges from 0 to 9) of 5 or greater indicates that a cohort or case-control study is reliable and of good quality. The Agency for Healthcare Research and Quality (AHRQ) is used for assessing the quality of cross-sectional studies and includes 11 items. If any research study reported epidemiological data in two difference countries, it was regarded as two studies in this report.

The prevalence of type 2 diabetes in patients with NAFLD or MAFLD (i.e., the number of cases divided by the study sample size) served as the primary outcome. If necessary, data for the missing cases were imputed using reported prevalence or incidence (%) as well as sample size. In addition, the incidence density of type 2 diabetes among patients with NAFLD or MAFLD were calculated. To estimate the pooled prevalence, the prevalence rates were combined in random-effects meta-analyses (normal-normal model) that took into account inter-study heterogeneity. Moreover, meta-analysis was performed using logit transformed proportions to obtain better statistical properties. Restricted maximum likelihood estimation was employed to determine inter-study heterogeneity using the Q (a significant Q statistic indicates moderators should be explored) and I^2^-statistic (% of total variability due to heterogeneity; values of ≥75% indicate heterogeneity), as well as by comparing results from studies grouped according to study-level characteristics (sex, publication year, region, country, average age, obese status, NAFLD diagnosis, and quality grade). To address the potential for misestimation due to liver index and hospital records of NAFLD definition, a sensitivity analysis was performed after excluding those studies, based on previous reports. Due to scientific objections regarding the assessment of study quality and the lack of essential data in the included studies (such as sampling frame, method, and representativeness of the general population), lower-quality studies were not excluded as sensitivity analyses. Instead, a mixed-effects model was used to conduct a meta-regression analysis to further explore the diversity between different findings. The percentage of males, average age of the sample population, geographic region, diagnostic method, follow-up duration, and publication year were examined both in univariate and multivariate meta-regression models. The Knapp and Hartung adjustments were used to test model coefficients, while the Holm’s method was employed to carry out pairwise comparisons for categorical moderators. A funnel plot, Begg-Mazumdar’s rank correlation test, and Egger’s regression test were used to estimate whether there were any publication or related biases.

In addition, Joinpoint Regression Analysis was used to calculate the average annual percent change (AAPC) and the corresponding 95% CIs in the temporal trend of each part with natural log-transformed rates. This indicator can reflect the average annual percentage change over a period of multiple years. The trends in rate were fitted with range from 0 to 5 joinpoints, and the best-fitting models was selected using the Monte Carlo permutation method.
