# Supplementary material for: Global epidemiology of type 2 diabetes in patients with NAFLD or MAFLD: a systematic review and meta-analysis
Source: BMC Med. 2024 Mar 6;22:101. doi: 10.1186/s12916-024-03315-0 (PMC10919055; doi:10.1186/s12916-024-03315-0)
Supplement: Supplementary file 2 — Additional file 2: Figures S1-S6. Fig. S1. Flow chart. Fig. S2. The prevalence of type 2 diabetes among patients with NAFLD-stratified by publication year. Fig. S3. The prevalence of type 2 diabetes among patients with MAFLD across 12 countries. Fig. S4. The prevalence of type 2 diabetes among patients with MAFLD-stratified by age, region, publication year, sample size and quality grade. Fig. S5. The incidence density of type 2 diabetes among patients with NAFLD-stratified by publication year. Fig. S6. Grading of Recommendation, Assessment, Development, and Evaluation (GRADE) instrument. [file 12916_2024_3315_MOESM2_ESM.docx]

**Additional file 2: Figures**

**Identification**

**Screening**

20,017 screened

1,901 full-text records assessed for eligibility

6,114 duplicates removed

18,116 excluded based on title or abstract

26,131 records identified

1,518 on Embase; 2,686 on PubMed

13,426 on Web of science; 8,501 on Medline

395 full-text records assessed for eligibility

1,506 excluded

1,361 did not report prevalence, incidence

density or have enough data to calculate

estimates

23 was teenager populations

14 not written in English

51 used the same database as a study already

included

364 records for NAFLD populations

340 records on prevalence study

35 records on incidence density study

42 records for MAFLD populations

40 records on prevalence study

3 records on incidence density study

**Analysis**

**Fig. S1. Flow chart.** DM, diabetes mellitus; MAFLD, metabolic associated fatty liver disease; NAFLD, Non-alcoholic fatty liver disease.

**Fig. S2. The prevalence of type 2 diabetes among patients with NAFLD-stratified by publication year.** CI, confidence interval; NAFLD, non-alcoholic fatty liver disease.


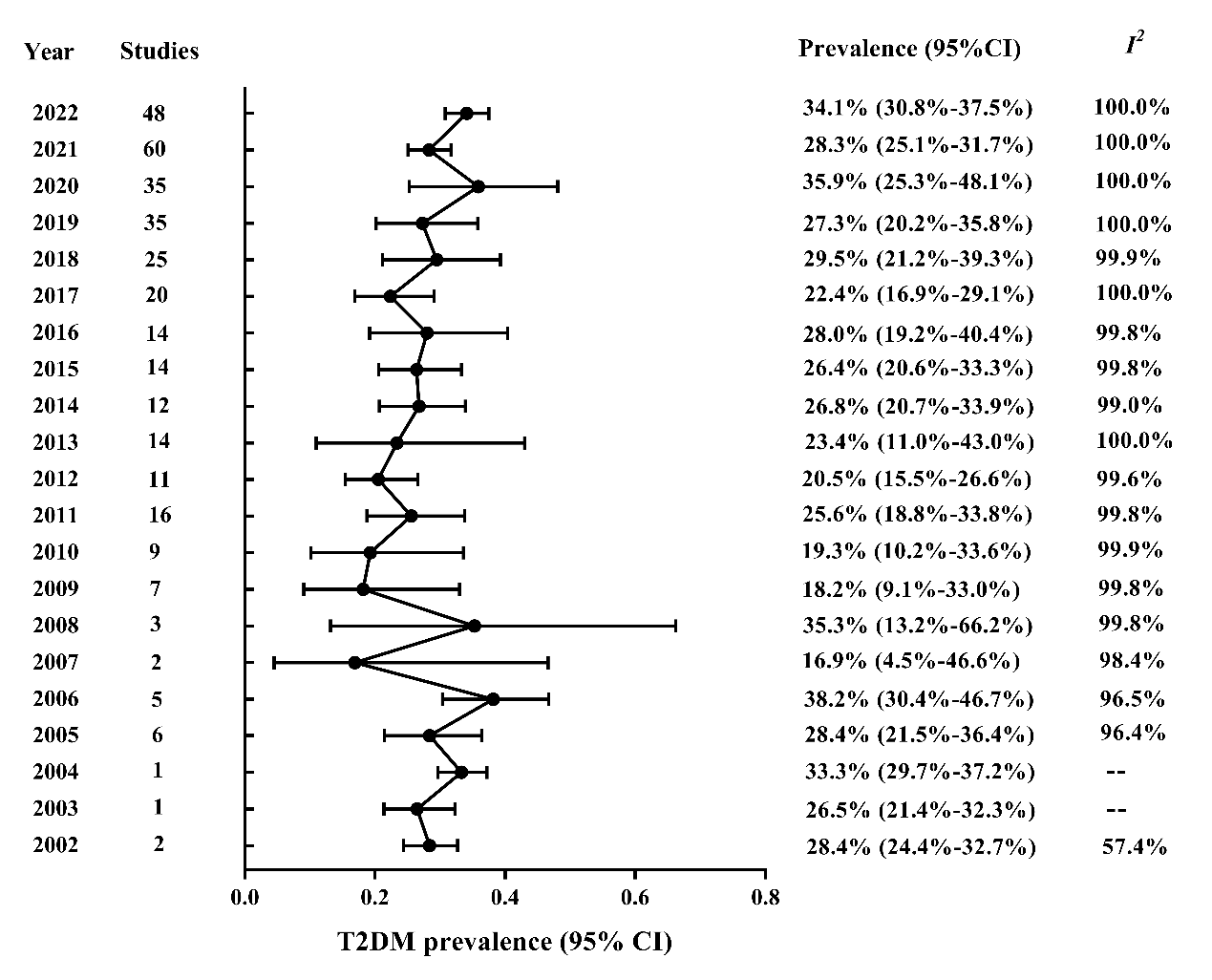


**The prevalence of type 2 diabetes (95% CI)**

**Fig. S3. The prevalence of type 2 diabetes among patients with MAFLD across 12 countries.** CI, confidence interval; NAFLD, non-alcoholic fatty liver disease.


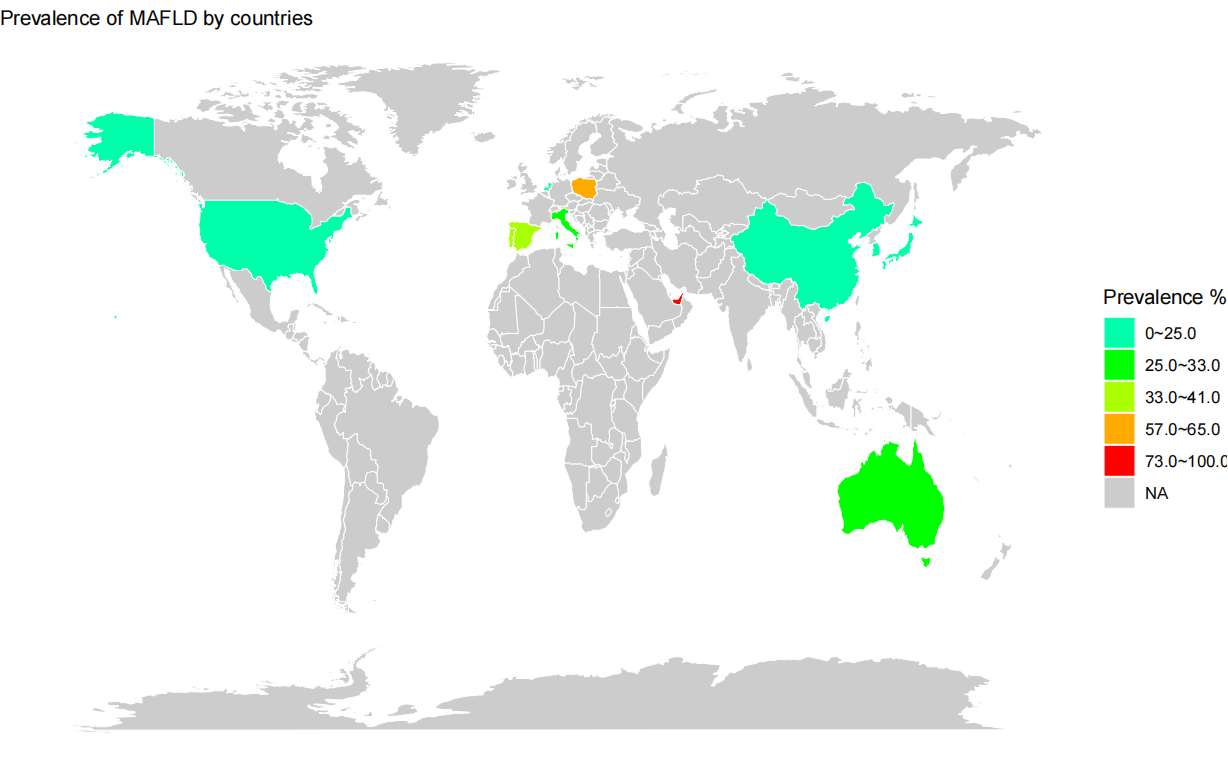


**
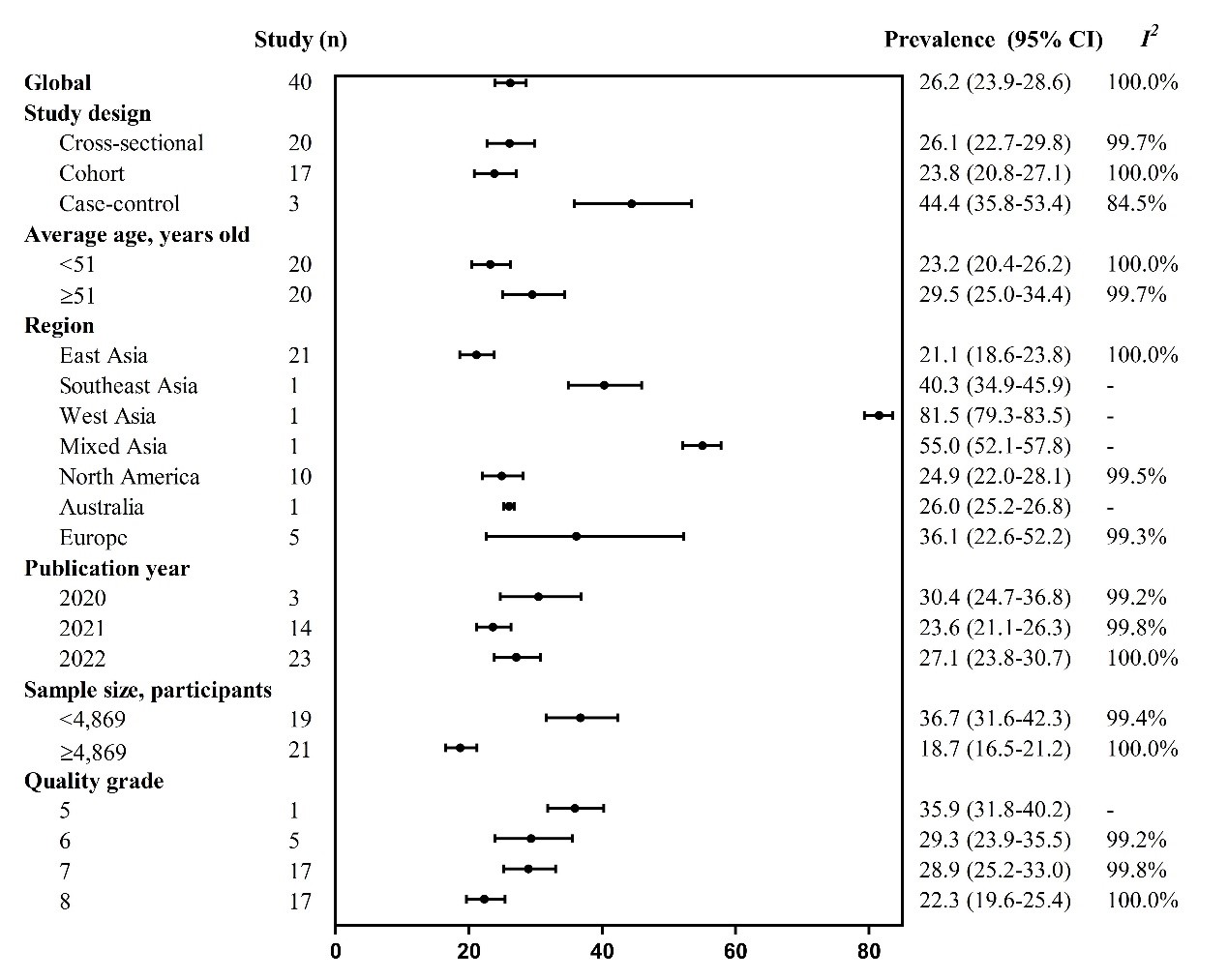
Fig. S4. The prevalence of type 2 diabetes among patients with MAFLD-stratified by age, region, publication year, sample size and quality grade.** CI, confidence interval; MAFLD, metabolic associated fatty liver disease.

**Fig. S5. The incidence density of type 2 diabetes among patients with NAFLD-stratified by publication year.** CI, confidence interval; NAFLD, non-alcoholic fatty liver disease.


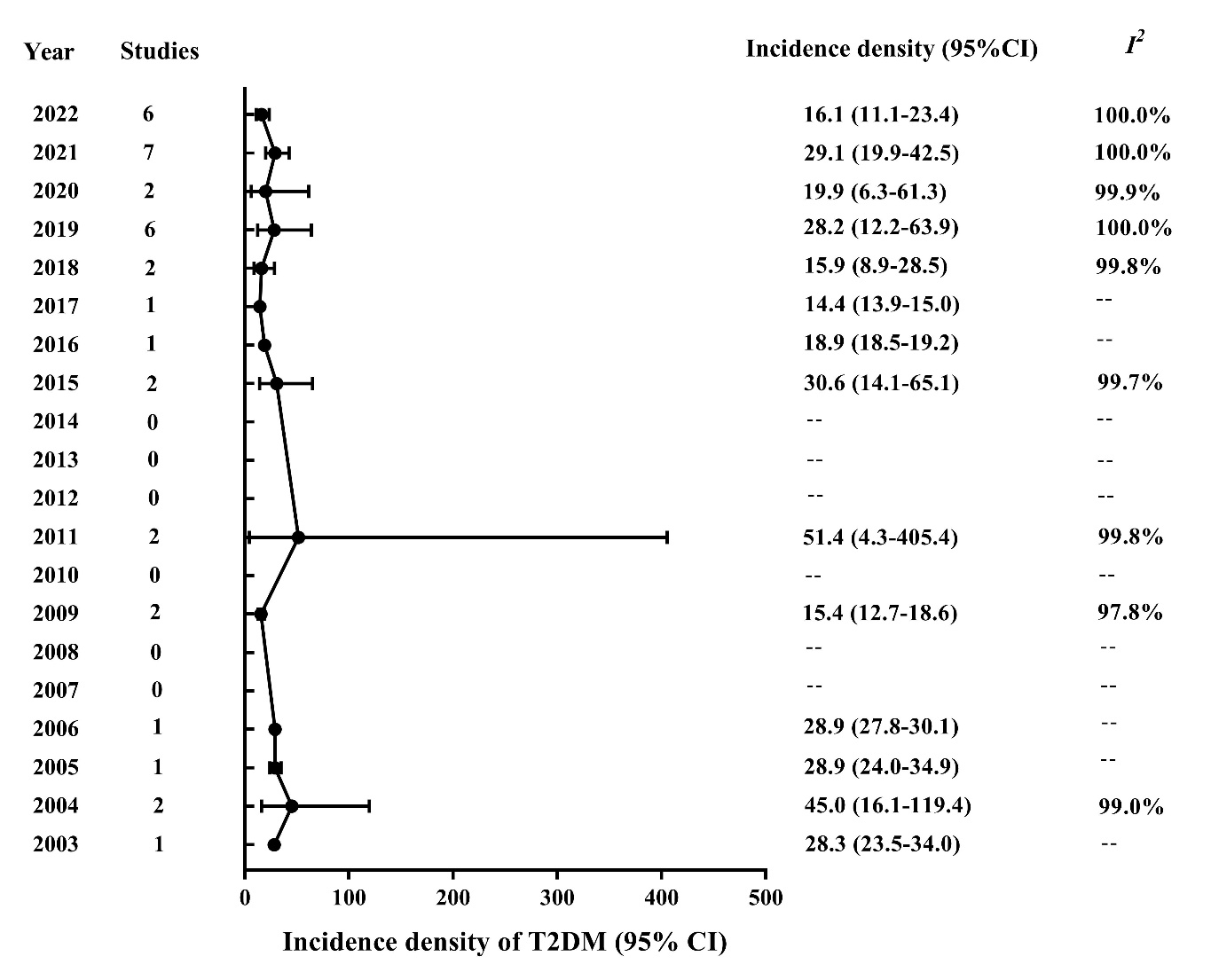


**Incidence density of type 2 diabetes (95% CI)**

**Fig. S6. Grading of Recommendation, Assessment, Development, and Evaluation (GRADE) instrument**


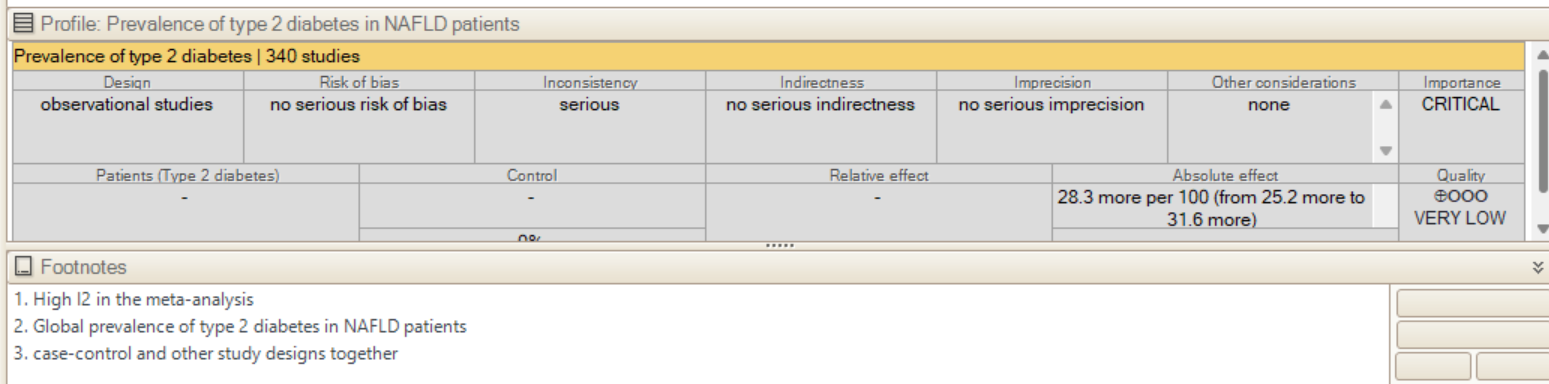

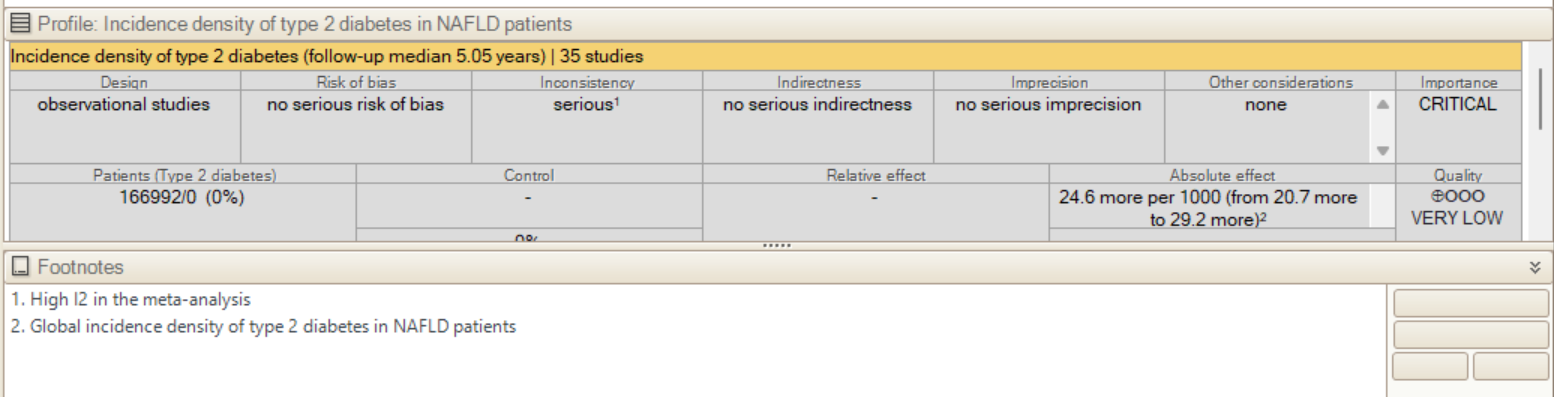

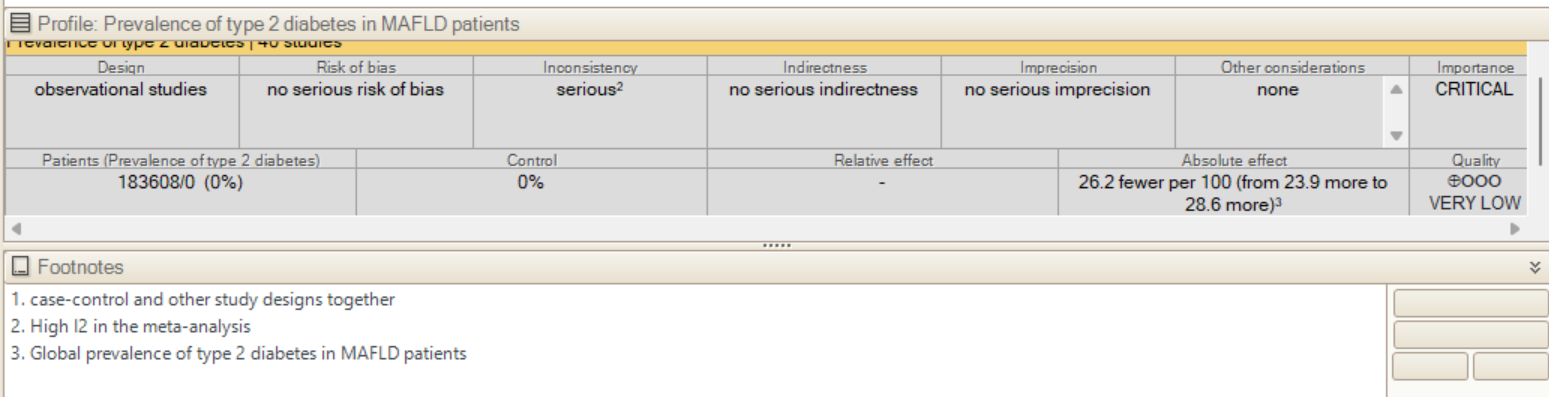

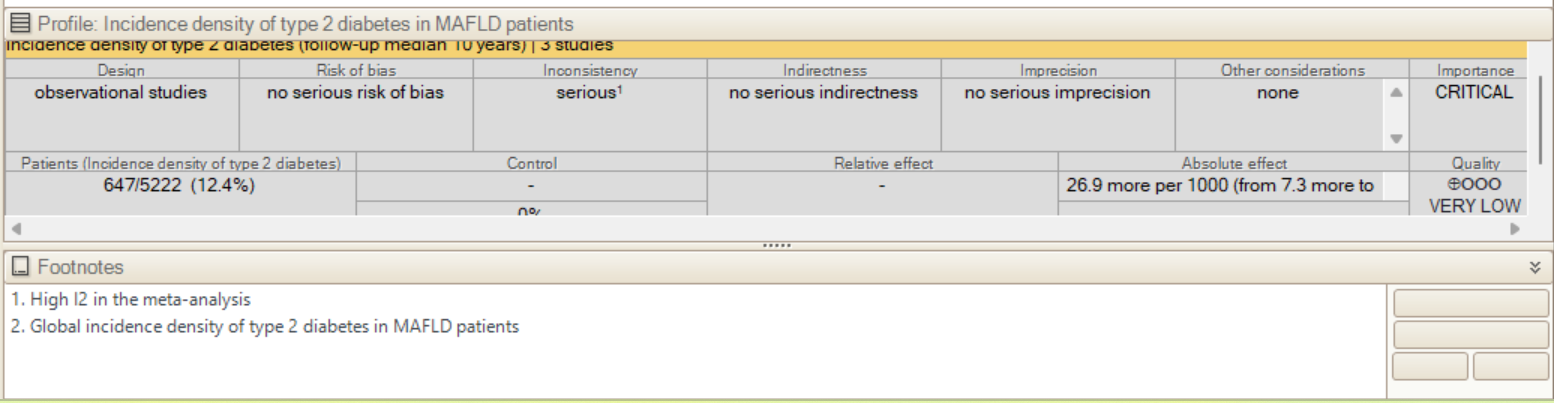


**GRADE Working Group grades of evidence**

High quality: We are very confident that the true effect lies close to that of the estimate of the effect

Moderate quality: We are moderately confident in the effect estimate. The true effect is likely to be close to the estimate of the effect, but there is a possibility that it is substantially different

Low quality: Our confidence in the effect estimate is limited. The true effect may be substantially different from the estimate of the effect

Very low quality: We have very little confidence in the effect estimate. The true effect is likely to be substantially different from the estimate of the effect.
