# Supplementary material for: Global epidemiology of type 2 diabetes in patients with NAFLD or MAFLD: a systematic review and meta-analysis
Source: BMC Med. 2024 Mar 6;22:101. doi: 10.1186/s12916-024-03315-0 (PMC10919055; doi:10.1186/s12916-024-03315-0)
Supplement: Supplementary file 3 — Additional file 3: Tables S1-S18. Table S1. Characteristics of the included studies in the prevalence of type 2 diabetes among NAFLD patients. Table S2. Characteristics of studies reporting the prevalence of type 2 diabetes in patients with NAFLD: source of heterogeneity. Table S3. Average annual percent change (%) in the prevalence of type 2 diabetes among NAFLD populations globally and by region. Table S4. The prevalence of type 2 diabetes among patients with NAFLD-stratified by age, region, publication year, sample size and diagnosis of NAFLD (excluding index and hospital record of NAFLD). Table S5. Secondary analysis of the prevalence of type 2 diabetes among patients with NAFLD. Table S6. Univariable and multivariable meta-regression analyses on the prevalence of type 2 diabetes among patients with NAFLD. Table S7. Characteristics of the included studies in the prevalence of type 2 diabetes among MAFLD patients. Table S8. Characteristics of studies reporting the prevalence of type 2 diabetes in patients with MAFLD: source of heterogeneity. Table S9. Average annual percent change (%) in the prevalence of type 2 diabetes among MAFLD populations globally and by region. Table S10. Univariable and multivariable meta-regression analyses on the prevalence of type 2 diabetes among patients with MAFLD. Table S11. Characteristics of the included studies in the incidence density of type 2 diabetes among NAFLD patients. Table S12. Characteristics of studies reporting the incidence density of type 2 diabetes in patients with NAFLD: source of heterogeneity. Table S13. The incidence density of type 2 diabetes among patients with NAFLD-stratified by age, region, publication year, sample size and diagnosis of NAFLD (excluding index and hospital record of NAFLD). Table S14. Average annual percent change (%) in the incidence density of type 2 diabetes among NAFLD populations globally and by region. Table S15. Univariable and multivariable meta-regression analyses on the incidence [file 12916_2024_3315_MOESM3_ESM.docx]

**Additional file 3: Tables S1-S18**

**Table S1. Characteristics of the included studies in the prevalence of type 2 diabetes among NAFLD patients.**

| Author | Country | Study design | Study year | Publication year | Male (%) | Average age, years | Sample size | Population of NAFLD | Events | Diagnosis of NAFLD | Covid-19 | Quality grade |
| --- | --- | --- | --- | --- | --- | --- | --- | --- | --- | --- | --- | --- |
| Masahiko Shimad *et al* | Japan | Cross-sectional | 1990-2001 | 2002 | 49.0 | 54 | 81 | 81 | 25 | Liver biopsy | No | 8 |
| Yi Wen Shi *et al* | China | Cross-sectional | 2009-2019 | 2002 | 30.4 | 48.9 | 158 | 158 | 42 | Mixed diagnostic methods | No | 6 |
| Krikor Kichian *et al* | Canada | Cross-sectional | 1996-2000 | 2003 | 51.0 | 46.4 | 49 | 49 | 13 | Liver biopsy | No | 5 |
| Zobair M. Younossi *et al* | USA | Cohort | 1979-1987 | 2004 | 48.0 | 53.05 | 157 | 132 | 44 | Liver biopsy | No | 6 |
| Eugene Han *et al* | Korea | Cross-sectional | 2008-2011 | 2022 | 39.6 | 54.6 | 1,795 | 234 | 84 | Index | No | 7 |
| Ibrahim Halil Bahcecioglu *et al* | Turkey | Cross-sectional | .. | 2005 | 76.0 | 38 | 93 | 93 | 17 | Liver biopsy | No | 6 |
| Akitaka Nonomura *et al* | Japan | Cross-sectional | 1999-2004 | 2005 | 53.9 | 49.7 | 76 | 76 | 29 | Liver biopsy | No | 6 |
| Janus P. Ong *et al* | USA | Cohort | .. | 2005 | 20.3 | 41.6 | 197 | 197 | 49 | Liver biopsy | No | 7 |
| Harminder Singh *et al* | Canada | Cross-sectional | 2002 | 2005 | 47.4 | 52.3 | 190 | 190 | 42 | Mixed diagnostic methods | No | 7 |
| Trang VoPham *et al* | USA | Cross-sectional | 2001-2011 | 2022 | 41.6 | 48.6 | 45,433,392 | 269,705 | 109,602 | Hospital record | No | 7 |
| Claire E. Thomas *et al* | USA | Cohort | 2004-2018 | 2022 | 43.2 | 57 | 27,834 | 27,834 | 7,356 | Hospital record | No | 6 |
| Maria Luiza Rodrigues Pereira Lima *et al* | Brazil | Cross-sectional | 2000-2003 | 2005 | 25.9 | 39.59 | 112 | 111 | 31 | Liver biopsy | No | 7 |
| Shiobhan R. Weston *et al* | USA | Cross-sectional | 1998-2000 | 2005 | 51.0 | 49.4 | 742 | 149 | 65 | Mixed diagnostic methods | No | 8 |
| Janani Arun *et al* | USA | Cohort | 2002-2005 | 2006 | 15.9 | 40.92 | 365 | 365 | 104 | Liver biopsy | No | 7 |
| Vincent Wai-Sun Wong *et al* | China | Cross-sectional | .. | 2006 | 57.0 | 45 | 121 | 80 | 46 | Liver biopsy | No | 8 |
| V. W. S. Wong *et al* | China | Cross-sectional | .. | 2006 | 60.5 | 45.41 | 124 | 124 | 51 | Liver biopsy | No | 7 |
| Thomas R. Riley *et al* | USA | Case-control | 1996-2002 | 2006 | 44.0 | 50.6 | 159 | 84 | 29 | Mixed diagnostic methods | No | 7 |
| Jian-Gao Fan *et al* | China | Cohort | 1995-2002 | 2006 | 90.5 | 39.38 | 1,146 | 358 | 115 | Hepatic ultrasonography | No | 7 |
| Muhammad Khurram *et al* | Pakistan | Cross-sectional | 2005-2006 | 2007 | 34.0 | 40.7 | 50 | 50 | 15 | Liver biopsy | No | 5 |
| Lei Zhang *et al* | China | Cross-sectional | 2004 | 2007 | 88.1 | 46 | 202 | 68 | 6 | Hepatic ultrasonography | No | 6 |
| S A Harrison *et al* | USA | Cohort | 2001-2005 | 2008 | 49.0 | 49 | 827 | 827 | 289 | Liver biopsy | No | 8 |
| Mark Anthony A De Lusong *et al* | Philippine | Cohort | 1999-2004 | 2008 | 35.1 | 42.2 | 1,102 | 134 | 92 | Mixed diagnostic methods | No | 7 |
| Noreen Hossain *et al* | USA | Cross-sectional | .. | 2009 | 22.9 | 43.6 | 432 | 432 | 25 | Liver biopsy | No | 6 |
| Erika R. Bjørnå *et al* | Norway | Cross-sectional | .. | 2022 | 36.7 | 52.1 | 47,927 | 2,373 | 349 | Index | No | 7 |
| Sangeeta R. Kashyap *et al* | USA | Cross-sectional | 2005-2007 | 2009 | 21.0 | 49 | 142 | 99 | 40 | Liver biopsy | No | 6 |
| Ki Chul Sung *et al* | Korea | Cohort | 1994-2003 | 2009 | 59.6 | 41 | 56,249 | 4,248 | 289 | Hepatic ultrasonography | No | 7 |
| James Frith *et al* | UK | Cohort | 2005-2007 | 2009 | 74.4 | 50 | 351 | 351 | 132 | Mixed diagnostic methods | No | 7 |
| M. Mahtab *et al* | Bangladesh | Cross-sectional | .. | 2009 | 59.6 | .. | 52 | 52 | 7 | Mixed diagnostic methods | No | 6 |
| Naim Abu-Freha *et al* | Israel | Cohort | 2000-2021 | 2022 | 52.8 | 59.5 | 211,955 | 211,955 | 49,263 | Hospital record | No | 7 |
| Nila Rafiq *et al* | USA | Cohort | .. | 2009 | 37.7 | 50.2 | 173 | 173 | 50 | Liver biopsy | No | 8 |
| Brent A. Neuschwander-Tetri *et al* | USA | Cross-sectional | 2004-2008 | 2010 | 36.0 | 50 | 1,266 | 1,266 | 31 | Liver biopsy | No | 8 |
| Iliana Doycheva *et al* | USA | Cross-sectional | 2002-2017 | 2019 | 77.1 | 57.7 | 24,141 | 1,925 | 1,193 | Hospital record | No | 6 |
| Jeong-Hoon Lee *et al* | Korea | Cross-sectional | 2006 | 2010 | 70.0 | 52.2 | 10,724 | 2,680 | 355 | Hepatic ultrasonography | No | 7 |
| Stuart McPherson *et al* | UK | Cross-sectional | 2003-2009 | 2010 | 61.0 | 51 | 145 | 145 | 73 | Liver biopsy | No | 6 |
| K. Gokulakrishnan *et al* | India | Cross-sectional | .. | 2010 | 49.2 | 46 | 193 | 72 | 19 | Hepatic ultrasonography | No | 6 |
| Chuan-Chuan Liu *et al* | China | Cross-sectional | 2003-2007 | 2010 | 64.0 | 44.5 | 7,204 | 840 | 43 | Hepatic ultrasonography | No | 7 |
| Jayani Manchanayake *et al* | Australia | Cohort | 2006-2009 | 2010 | 54.0 | 53 | 111 | 111 | 39 | Hepatic ultrasonography | No | 7 |
| G. Bianchi *et al* | Italy | Cross-sectional | .. | 2010 | .. | 78 | 1,144 | 486 | 68 | Hepatic ultrasonography | No | 6 |
| Mohamed E. E. Shams *et al* | Egypt | Cross-sectional | .. | 2011 | 62.9 | 53.3 | 105 | 105 | 47 | Mixed diagnostic methods | No | 6 |
| Nadege T. Touzin *et al* | USA | Cohort | 2007-2010 | 2011 | 51.5 | 54.7 | 233 | 94 | 26 | Hepatic ultrasonography | No | 8 |
| Hüseyin Saadettin Uslusoy *et al* | Turkey | Cross-sectional | .. | 2011 | 49.4 | 47.9 | 81 | 70 | 20 | Hepatic ultrasonography | No | 6 |
| Akinobu Nakamura *et al* | Japan | Cross-sectional | 2004-2010 | 2011 | 54.4 | 51.4 | 147 | 147 | 36 | Liver biopsy | No | 7 |
| Luca Valenti *et al* | Italy | Case-control | 1999-2008 | 2011 | 53.2 | 48 | 758 | 758 | 261 | Liver biopsy | No | 8 |
| Sven H. Loosen *et al* | Germany | Cohort | 2000-2015 | 2021 | 50.0 | 59.5 | 101,378 | 50,689 | 24,381 | Hospital record | No | 6 |
| Toshiya Machida *et al* | Japan | Cross-sectional | 2013-2020 | 2022 | .. | 52.57 | 7,716,908 | 9,113 | 5,631 | Hospital record | No | 6 |
| Donghee Kim *et al* | Korea | Cross-sectional | 2003-2008 | 2011 | 60.7 | 57.5 | 4,023 | 1,617 | 276 | Hepatic ultrasonography | No | 7 |
| Christopher D. Williams *et al* | USA | Cohort | .. | 2011 | 49.1 | 54.6 | 328 | 151 | 40 | Mixed diagnostic methods | No | 8 |
| Helma P. Cotrim *et al* | Brazil | Cross-sectional | .. | 2011 | 53.3 | 49.68 | 1,280 | 1,280 | 291 | Hepatic ultrasonography | No | 6 |
| Olubunmi Oladunjoye *et al* | USA | Cohort | 2010-2014 | 2021 | 40.5 | 55.2 | 953,598 | 194,787 | 94,082 | Hospital record | No | 7 |
| James H. Tabibian *et al* | USA | Cross-sectional | 1998-2008 | 2011 | 50.0 | 49 | 90 | 90 | 34 | Liver biopsy | No | 6 |
| Claudia O. Zein *et al* | USA | Cross-sectional | 2004-2008 | 2011 | 63.0 | 49 | 1,091 | 1,091 | 318 | Liver biopsy | No | 8 |
| Ilaria Barchetta *et al* | Italy | Cross-sectional | .. | 2011 | 53.4 | 52.07 | 262 | 162 | 63 | Hepatic ultrasonography | No | 6 |
| Natasha Chandok *et al* | Canada | Cohort | 1995-2010 | 2011 | 47.1 | 46.9 | 482 | 482 | 83 | Mixed diagnostic methods | No | 6 |
| Sun Ling *et al* | China | Cross-sectional | 2008-2009 | 2011 | 65.3 | 62 | 542 | 248 | 66 | CT | No | 6 |
| Shumei He *et al* | China | Cross-sectional | 2007 | 2011 | 51.0 | .. | 6,043 | 605 | 180 | Hepatic ultrasonography | No | 8 |
| S. Francque *et al* | Belgium | Cross-sectional | 2004 | 2011 | 54.0 | 49.4 | 230 | 230 | 37 | Liver biopsy | No | 6 |
| Yusuke Kawamura *et al* | Japan | Cohort | 1997-2010 | 2011 | .. | 49 | 6,508 | 6,508 | 536 | Hepatic ultrasonography | No | 8 |
| Raj Vuppalanchi • Robert J *et al* | USA | Cross-sectional | .. | 2012 | 36.0 | 49 | 864 | 864 | 240 | Liver biopsy | No | 7 |
| Kiran Bambha *et al* | USA | Cross-sectional | 2004-2008 | 2012 | 32.0 | 50 | 1,026 | 628 | 235 | Liver biopsy | No | 6 |
| Xinrong Zhang *et al* | China | Cohort | 2000-2014 | 2021 | 45.4 | 56.2 | 8,351 | 8,351 | 4,968 | Hospital record | No | 7 |
| Menha Swellam *et al* | Egypt | Case-control | .. | 2012 | 46.7 | 48 | 120 | 90 | 43 | Liver biopsy | No | 8 |
| Zobair M. Younossi *et al* | USA | Cohort | 1988-1994 | 2012 | .. | 43.29 | 11,613 | 2,492 | 361 | Hepatic ultrasonography | No | 6 |
| Jonas Valantinas *et al* | Lithuania | Cross-sectional | 2008-2009 | 2012 | 37.3 | 33.1 | 798 | 798 | 175 | Mixed diagnostic methods | No | 6 |
| Abhishak Chandra Gupta *et al* | India | Case-control | 2007-2009 | 2012 | 68.0 | 36.5 | 137 | 137 | 17 | Liver biopsy | No | 7 |
| Anna Ludovica Fracanzani *et al* | Italy | Cross-sectional | / | 2012 | 71.2 | 48.43 | 524 | 524 | 55 | Liver biopsy | No | 7 |
| Erifili Hatziagelaki *et al* | Greece | Cohort | 2006-2009 | 2012 | 49.6 | 50.1 | 152 | 152 | 29 | Hepatic ultrasonography | No | 7 |
| Shi Lei *et al* | China | Cross-sectional | 2008-2009 | 2012 | 50.7 | 54.5 | 9,378 | 968 | 112 | Hepatic ultrasonography | No | 7 |
| Waleed Al-hamoudi *et al* | Saudi Arabia | Cohort | 2009 | 2012 | 51.0 | 44.7 | 1,312 | 202 | 69 | Hepatic ultrasonography | No | 6 |
| Seok-Chin Goh *et al* | Malaysia | Cross-sectional | 2000-2009 | 2013 | 33.7 | 46 | 1,612 | 368 | 66 | Hepatic ultrasonography | No | 8 |
| Ramesh Kumar *et al* | India | Cross-sectional | 2009-2011 | 2013 | 82.7 | 40.54 | 336 | 205 | 43 | Mixed diagnostic methods | No | 6 |
| Niraj S. Patel *et al* | USA | Case-control | .. | 2013 | 38.0 | 45.6 | 92 | 43 | 15 | Liver biopsy | No | 6 |
| Gary A. Abrams *et al* | USA | Case-control | 2002-2003 | 2013 | 11.8 | 40.8 | 195 | 71 | 40 | Liver biopsy | No | 8 |
| Yeonjung Ha *et al* | Korea | Cross-sectional | 2013-2015 | 2017 | 47.3 | 44.8 | 1,812 | 409 | 90 | Index | No | 7 |
| Srinevas K. Reddy *et al* | Mixed countries | Cohort | 1991-2011 | 2013 | 46.4 | 63 | 181 | 31 | 12 | Liver biopsy | No | 7 |
| Motoi Hashiba *et al* | Japan | Cross-sectional | .. | 2013 | 59.8 | 47.99 | 169 | 169 | 52 | Liver biopsy | No | 6 |
| Alireza Bakhshipour *et al* | Iran | Cross-sectional | 2008-2010 | 2013 | 68.5 | 37.4 | 403 | 403 | 48 | Mixed diagnostic methods | No | 6 |
| Mohammad Ebrahim Ghamar Chehreh *et al* | Iran | Cross-sectional | 2009 | 2013 | 60.6 | 45.9 | 528 | 528 | 57 | Hepatic ultrasonography | No | 6 |
| Maria Stepanova *et al* | USA | Cohort | .. | 2013 | 39.4 | 50.3 | 289 | 289 | 75 | Liver biopsy | No | 6 |
| Whye Lian Cheah *et al* | Malaysia | Cross-sectional | .. | 2013 | 46.8 | 48.4 | 77 | 34 | 8 | Hepatic ultrasonography | No | 7 |
| Shira Zelber-Sagi *et al* | Israel | Cohort | .. | 2013 | 50.4 | 48.78 | 141 | 35 | 3 | Hepatic ultrasonography | No | 7 |
| Yoosoo Chang *et al* | Korea | Cross-sectional | 2005-2006 | 2013 | 62.5 | 37.47 | 43,166 | 11,652 | 549 | Hepatic ultrasonography | No | 7 |
| Takashi Nakahara *et al* | Japan | Cohort | 2001-2012 | 2013 | 51.9 | 51 | 1,365 | 1,365 | 646 | Liver biopsy | No | 8 |
| Hideyuki Hyogo *et al* | Japan | Cross-sectional | .. | 2014 | 34.7 | 55.7 | 194 | 194 | 97 | Mixed diagnostic methods | No | 7 |
| Paul Angulo *et al* | Mixed countries | Cohort | .. | 2014 | 57.8 | 46.9 | 1,014 | 1,014 | 298 | Liver biopsy | No | 8 |
| Rohit Loomba *et al* | USA | Cohort | 2007-2015 | 2020 | 40.0 | 67.62 | 10,826,456 | 446,357 | 244,960 | Hospital record | No | 8 |
| Yan Li *et al* | China | Cross-sectional | 2009-2012 | 2014 | 55.7 | 48.1 | 325 | 215 | 60 | Liver biopsy | No | 7 |
| Edoardo Alessandro Pulixi *et al* | Italy | Cross-sectional | 2009-2013 | 2014 | 75.0 | 49.9 | 239 | 159 | 46 | Liver biopsy | No | 9 |
| M. Del Ben *et al* | Italy | Cross-sectional | .. | 2014 | 64.6 | 54.3 | 209 | 209 | 65 | Hepatic ultrasonography | No | 7 |
| Juan Ybarra *et al* | Spain | Cross-sectional | 2012 | 2014 | 87.4 | 37.86 | 151 | 151 | 21 | Hepatic ultrasonography | No | 6 |
| Roberto Catanzaro *et al* | Italy | Cross-sectional | 2012-2013 | 2014 | 40.1 | 56 | 389 | 206 | 60 | Hepatic ultrasonography | No | 7 |
| Gayatri Pemmasani *et al* | USA | Cohort | 2016 | 2020 | 38.4 | 61.5 | 41,005 | 41,005 | 25,587 | Hospital record | No | 7 |
| Y.-L. Liu *et al* | UK | Case-control | .. | 2014 | 64.8 | 56.07 | 375 | 375 | 185 | Liver biopsy | No | 8 |
| Emad A Rakha *et al* | UK | Case-control | 1991-2001 | 2014 | 73.0 | 48.6 | 360 | 206 | 42 | Liver biopsy | No | 7 |
| Giordano Gianotti *et al* | Italy | Cohort | 2003 | 2014 | 48.0 | 69.5 | 171 | 62 | 8 | Hepatic ultrasonography | No | 6 |
| Ren-Nan Feng *et al* | China | Cross-sectional | 2012-2013 | 2014 | 75.0 | 45.73 | 1,779 | 898 | 147 | Hepatic ultrasonography | No | 7 |
| Gabriel Costa de Andrade *et al* | Brazil | Cross-sectional | 2006-2007 | 2014 | 24.0 | 50.25 | 62 | 60 | 17 | Mixed diagnostic methods | No | 7 |
| Lourianne Nascimento Cavalcante *et al* | Brazil | Cross-sectional | .. | 2015 | 33.6 | 56 | 131 | 131 | 79 | Liver biopsy | No | 5 |
| Lourianne Nascimento Cavalcante  *et al* | Portugal | Cross-sectional | .. | 2015 | 22.2 | 47 | 90 | 90 | 42 | Liver biopsy | No | 5 |
| Bimal Chandra Shi *et al* | Bangladesh | Cross-sectional | 2011-2012 | 2015 | 54.6 | 36.2 | 216 | 216 | 38 | Hepatic ultrasonography | No | 6 |
| Chi-Sheng Hung *et al* | China | Cross-sectional | 2005-2006 | 2015 | 51.5 | 50.1 | 31,116 | 12,924 | 2,128 | Hepatic ultrasonography | No | 7 |
| Hajime Yamazaki *et al* | Japan | Cohort | 2000-2012 | 2015 | 58.8 | 43.8 | 4,604 | 728 | 117 | Hepatic ultrasonography | No | 7 |
| Chia-Chu Chang *et al* | China | Cross-sectional | 2012-2014 | 2015 | 52.6 | 53.5 | 133 | 90 | 42 | MRI | No | 7 |
| Michelle T. Long *et al* | USA | Cross-sectional | 2002-2005 | 2015 | 48.6 | 52 | 2,284 | 350 | 52 | CT | No | 7 |
| Min-Sun Kwak *et al* | Korea | Cross-sectional | 2010 | 2015 | 49.3 | 48.5 | 17,612 | 5,337 | 498 | Hepatic ultrasonography | No | 8 |
| Palanivelu Praveenraj *et al* | India | Cohort | 2008-2009 | 2015 | 39.3 | 45.25 | 134 | 88 | 47 | Liver biopsy | No | 6 |
| Aki J. Käräjämäki *et al* | Finland | Cohort | 2017-2020 | 2015 | 47.0 | 51.26 | 958 | 249 | 69 | Hepatic ultrasonography | No | 7 |
| Ahmed Abdel-Razik *et al* | Egypt | Cohort | 2006-2018 | 2021 | 31.4 | 48 | 188 | 94 | 32 | Index | No | 7 |
| Ilkay S. Idilman *et al* | Turkey | Cross-sectional | 2010-2011 | 2015 | 48.8 | 45.4 | 41 | 41 | 5 | MRI | No | 6 |
| Tingting Du *et al* | China | Cross-sectional | 2008-2010 | 2015 | .. | 49.66 | 10,761 | 4,318 | 495 | Hepatic ultrasonography | No | 8 |
| Ryosuke Tateishi *et al* | Japan | Cohort | 1991-2010 | 2015 | 75.5 | 72 | 5,326 | 596 | 359 | Hepatic ultrasonography | No | 7 |
| Jee-Fu Huang *et al* | China | Cohort | 2009-2014 | 2015 | 72.3 | 43 | 130 | 130 | 31 | Liver biopsy | No | 7 |
| Mavidi Sunil Kumar *et al* | India | Cross-sectional | .. | 2016 | 48.0 | 42.9 | 75 | 50 | 25 | Hepatic ultrasonography | No | 6 |
| Stefano Ballestri *et al* | Italy | Cross-sectional | 2001-2003 | 2016 | 72.0 | 47 | 118 | 118 | 30 | Mixed diagnostic methods | No | 8 |
| Serena Pelusi *et al* | Italy | Cohort | 1992-2015 | 2016 | 59.3 | 48 | 118 | 118 | 18 | Liver biopsy | No | 8 |
| Kuan-Ta Wu *et al* | China | Cross-sectional | 1999-2013 | 2016 | 64.0 | 43 | 44,767 | 24,160 | 2,079 | Hepatic ultrasonography | No | 7 |
| Elliot B. Tapper *et al* | Israel | Cohort | 2009-2014 | 2016 | 60.0 | 51.5 | 151 | 151 | 46 | Liver biopsy | No | 8 |
| Julia Uhanova *et al* | Canada | Cohort | .. | 2016 | 47.1 | 46.9 | 482 | 482 | 83 | Mixed diagnostic methods | No | 6 |
| George BB Goh *et al* | USA | Cross-sectional | .. | 2016 | 44.2 | 48 | 405 | 405 | 171 | Liver biopsy | No | 7 |
| Gordana Petrović *et al* | Saudi Arabia | Case-control | 2012-2014 | 2016 | 38.3 | 49.29 | 86 | 55 | 26 | Hepatic ultrasonography | No | 6 |
| Hannes Hagström *et al* | Sweden | Cohort | .. | 2016 | 69.0 | 55.9 | 120 | 120 | 49 | Liver biopsy | No | 7 |
| Fabio Conti *et al* | Italy | Cross-sectional | 2008-2009 | 2016 | 38.3 | 51 | 488 | 157 | 20 | Hepatic ultrasonography | No | 6 |
| Bo Kyung Koo *et al* | USA | Cohort | 2013 | 2016 | 46.9 | 53.05 | 309 | 240 | 95 | Liver biopsy | No | 7 |
| Anna Ludovica Fracanzani *et al* | Italy | Cross-sectional | 2013-2014 | 2016 | 61.3 | 59.3 | 512 | 503 | 79 | Mixed diagnostic methods | No | 7 |
| Sombat Treeprasertsuk *et al* | Thailand | Cross-sectional | 2009-2012 | 2016 | 47.0 | 40.9 | 139 | 139 | 53 | Mixed diagnostic methods | No | 7 |
| Vincent Wai-Sun Wong *et al* | China | Cohort | .. | 2016 | 70.8 | 63 | 612 | 356 | 147 | Hepatic ultrasonography | No | 8 |
| Laura E. Dichtel *et al* | USA | Cross-sectional | 2010-2015 | 2017 | 46.5 | 52 | 142 | 142 | 54 | Liver biopsy | No | 7 |
| Ueamporn Summart *et al* | Thailand | Cross-sectional | 2013-2015 | 2017 | 22.0 | 55.5 | 34,709 | 7,584 | 687 | Hepatic ultrasonography | No | 6 |
| Nobuyuki Toshikuni *et al* | Japan | Cross-sectional | 2009-2011 | 2017 | 58.4 | 54 | 753 | 581 | 302 | Liver biopsy | No | 6 |
| W.-K. Chan *et al* | Mixed countries | Cohort | Hong Kong:2006-2015 Malaysia:2012-2015 Thailand:2007-2014 Japan:2009-2016 China:2012-2013 Korea:2009-2016 Singapore:2002-2015 Indonesia:2011-2015 | 2017 | 56.4 | 47 | 1,008 | 1,008 | 559 | Liver biopsy | No | 8 |
| Eline H. van den Berg *et al* | Netherlands | Cross-sectional | .. | 2018 | 49.8 | 49.7 | 7,622 | 2,083 | 177 | Index | No | 7 |
| Xuerong Wen *et al* | USA | Cohort | 2010-2015 | 2021 | 52.3 | 67.47 | 39,317 | 430 | 168 | Hospital record | No | 7 |
| Hye Won Lee *et al* | Korea | Cross-sectional | 2013-2014 | 2017 | 58.2 | 54 | 2,749 | 1,178 | 150 | Hepatic ultrasonography | No | 7 |
| Salvatore Petta *et al* | Italy | Cross-sectional | 2004-2015 | 2017 | 67.9 | 61.5 | 863 | 863 | 356 | Liver biopsy | No | 8 |
| Akanksha Singh *et al* | India | Cross-sectional | .. | 2017 | 48.0 | 44 | 75 | 50 | 25 | Hepatic ultrasonography | No | 7 |
| StudyGalit Weinstein *et al* | USA | Cross-sectional | 2002-2011 | 2017 | 33.4 | 67 | 766 | 137 | 19 | CT | No | 8 |
| Reona Morio *et al* | Japan | Cohort | 2003-2012 | 2017 | 62.7 | 48.2 | 375 | 375 | 103 | Liver biopsy | No | 7 |
| Y. A. Patel *et al* | USA | Case-control | 2005-2015 | 2017 | 86.0 | 56.41 | 399 | 190 | 131 | Liver biopsy | No | 7 |
| M. Dallio *et al* | Italy | Case-control | .. | 2017 | 42.8 | 57 | 120 | 60 | 27 | Liver biopsy | No | 8 |
| Dae Won Jun *et al* | Korea | Cohort | 2000-2010 | 2017 | 70.7 | 36.4 | 412 | 328 | 109 | Liver biopsy | No | 7 |
| Seolhye Kim *et al* | Korea | Cohort | 2002-2014 | 2017 | 53.9 | 37.25 | 208,578 | 50,964 | 3,021 | Hepatic ultrasonography | No | 7 |
| Abbas Ali Tasneem *et al* | Pakistan | Cross-sectional | 2011-2017 | 2017 | 79.2 | 34.95 | 96 | 96 | 10 | Hepatic ultrasonography | No | 6 |
| Ji Cheol Bae *et al* | Korea | Cohort | 2005-2009 | 2017 | 68.9 | 44.6 | 7,849 | 2,292 | 227 | Hepatic ultrasonography | No | 7 |
| Sabry M. Abdeldyem *et al* | Egypt | Cross-sectional | 2013-2015 | 2017 | 55.0 | 65.41 | 200 | 85 | 53 | Hepatic ultrasonography | No | 7 |
| Soon Sun Kim *et al* | Korea | Cohort | 2000-2010 | 2017 | 57.9 | 60.5 | 2,920 | 924 | 61 | Hepatic ultrasonography | No | 7 |
| Hannes Hagstr€om *et al* | Sweden | Cohort | 1971-2009 | 2018 | 62.2 | 48.2 | 646 | 646 | 93 | Liver biopsy | No | 8 |
| H. Peng *et al* | USA | Cross-sectional | 2003-2004, 2005-2006 | 2022 | 56.8 | 51 | 2,294 | 969 | 139 | Index | No | 7 |
| Carolina Castro Porto Silva Janovsky *et al* | Israel | Cross-sectional | 2014-2015 | 2018 | 73.0 | 46.11 | 10,539 | 3,932 | 200 | Hepatic ultrasonography | No | 6 |
| Masato Yoneda *et al* | Japan | Cohort | 2013-2019 | 2021 | 62.4 | 46.2 | 3,995,637 | 142,158 | 28,743 | Index | No | 7 |
| Omer Shahab *et al* | USA | Cross-sectional | .. | 2018 | 52.8 | 47.78 | 2,566 | 2,566 | 391 | Hepatic ultrasonography | No | 7 |
| Preya Janubhai Patel *et al* | Australia | Cross-sectional | 2015-2017 | 2018 | 54.4 | 57.3 | 252 | 252 | 208 | Hepatic ultrasonography | No | 7 |
| Norio Akuta *et al* | Japan | Cohort | 1976-2017 | 2018 | 60.9 | 51 | 402 | 402 | 126 | Liver biopsy | No | 6 |
| Yu-Jin Kwon *et al* | Korea | Cross-sectional | 2007-2010 | 2018 | 50.0 | 45.5 | 7,681 | 2,458 | 157 | Hepatic ultrasonography | No | 8 |
| Shaoyou Qin *et al* | China | Cross-sectional | 2015-2017 | 2018 | 55.6 | 52.1 | 1,415 | 1,415 | 234 | Hepatic ultrasonography | No | 7 |
| Goh Eun Chung *et al* | Korea | Cross-sectional | 2011 | 2018 | 42.1 | 52.9 | 1,190 | 331 | 137 | Hepatic ultrasonography | No | 8 |
| Sampath De Silva *et al* | USA | Cross-sectional | 2010-2016 | 2018 | 69.5 | 47.74 | 175 | 169 | 68 | Liver biopsy | No | 7 |
| Bo Kyung Koo *et al* | Korea | Cross-sectional | 2013 | 2018 | 65.3 | 53 | 190 | 190 | 57 | Mixed diagnostic methods | No | 8 |
| Arianna Mazzotti *et al* | Italy | Cohort | 2010-2015 | 2018 | 53.5 | 51.6 | 716 | 716 | 238 | Hepatic ultrasonography | No | 8 |
| Takefumi Kimura *et al* | Japan | Cohort | 2003-2016 | 2018 | 45.2 | 55.69 | 301 | 301 | 110 | Liver biopsy | No | 7 |
| Christian Labenz *et al* | Germany | Cohort | 2010 | 2018 | 52.5 | 51 | 261 | 261 | 78 | Liver biopsy | No | 8 |
| Bashu Dev Pardhe *et al* | Nepal | Cross-sectional | .. | 2018 | 52.4 | 56 | 429 | 219 | 36 | Hepatic ultrasonography | No | 7 |
| Antonino Tuttolomondo *et al* | Italy | Case-control | 2014-2015 | 2018 | .. | 52.68 | 163 | 80 | 31 | Liver biopsy | No | 7 |
| Haiyan Li *et al* | China | Cross-sectional | 2008-2017 | 2018 | 60.8 | 65.61 | 306 | 130 | 79 | Hepatic ultrasonography | No | 7 |
| Geraldine J. Ooi *et al* | Australia | Cohort | 2015-2017 | 2018 | 24.5 | 44.4 | 216 | 216 | 56 | Liver biopsy | No | 7 |
| Rocío Aller de la Fuente *et al* | Spain | Cross-sectional | 2012-2016 | 2018 | 52.5 | 43.8 | 217 | 217 | 36 | Liver biopsy | No | 7 |
| Fabiola Rabelo *et al* | Brazil | Cross-sectional | 2009-2012 | 2018 | 25.1 | 55.49 | 207 | 207 | 130 | Liver biopsy | No | 6 |
| Pradeep Nigam *et al* | India | Cross-sectional | 2016-2017 | 2018 | 40.0 | 48.99 | 200 | 200 | 117 | Hepatic ultrasonography | No | 7 |
| Hamid Reza Omrani *et al* | Iran | Cross-sectional | 2016 | 2018 | 62.7 | 63.68 | 150 | 30 | 20 | Hepatic ultrasonography | No | 7 |
| Stuart Gordon *et al* | USA | Cohort | 2007-2015 | 2019 | 40.0 | 67.7 | 255,681 | 255,681 | 144,941 | Hospital record | No | 8 |
| Abdus Saboor Shah *et al* | Pakistan | Cohort | 2013-2014 | 2018 | 49.4 | 55.8 | 164 | 78 | 21 | Hepatic ultrasonography | No | 6 |
| Hon-Jhe Chen *et al* | China | Cohort | 2000-2013 | 2018 | 65.2 | 44.94 | 21,590 | 4,318 | 818 | Hospital record | No | 6 |
| Shahinul Alam *et al* | Bangladesh | Cross-sectional | 2013-2016 | 2019 | 37.5 | 39.9 | 851 | 851 | 220 | Liver biopsy | No | 6 |
| Masahide Hamaguch *et al* | Japan | Cohort | 2003-2016 | 2019 | 58.9 | 43.87 | 27,944 | 3,211 | 214 | Hepatic ultrasonography | No | 6 |
| Abdul Sattar Arif Khammas *et al* | Malaysia | Cross-sectional | 2015-2016 | 2019 | 48.1 | 54.54 | 628 | 235 | 26 | Hepatic ultrasonography | No | 6 |
| Ahmad Moolla *et al* | UK | Cohort | 2015-2017 | 2019 | 64.2 | 53 | 165 | 165 | 97 | Mixed diagnostic methods | No | 6 |
| Oumarou Nabi *et al* | France | Cross-sectional | 2012-2018 | 2020 | 45.4 | 47.2 | 102,344 | 16,695 | 3,787 | Index | No | 8 |
| Takashi Shida *et al* | Japan | Cohort | 2011-2015 | 2019 | 39.1 | 55.5 | 92 | 92 | 39 | Hepatic ultrasonography | No | 7 |
| Peiling Tsou *et al* | USA | Cohort | .. | 2019 | 49.2 | 48.3 | 2,404 | 2,404 | 662 | Hepatic ultrasonography | No | 7 |
| Seishin Azuma *et al* | Japan | Case-control | 2000-2016 | 2019 | 47.3 | 66.97 | 182 | 182 | 79 | Mixed diagnostic methods | No | 6 |
| John William Blackett *et al* | Columbia | Cross-sectional | 2007-2017 | 2019 | 50.4 | 60.7 | 369 | 123 | 62 | Liver biopsy | No | 6 |
| Hyo Jung Cho *et al* | Korea | Cohort | 2000 | 2019 | 57.6 | 44.2 | 2,726 | 825 | 113 | Mixed diagnostic methods | No | 7 |
| Rola F. Jaafar *et al* | USA | Cross-sectional | 2016-2018 | 2019 | 62.1 | 53.69 | 248 | 248 | 73 | Hepatic ultrasonography | No | 6 |
| Donghee Kim *et al* | Korea | Cross-sectional | 2013-2018 | 2019 | 50.3 | 52.6 | 664 | 542 | 240 | Liver biopsy | No | 7 |
| Igor V Maev *et al* | Russia | Cross-sectional | 2015-2016 | 2019 | 37.8 | 48.7 | 2,843 | 2,843 | 477 | Mixed diagnostic methods | No | 6 |
| Brooks V. Udelsman *et al* | USA | Cross-sectional | 2001-2017 | 2019 | 28.4 | 46.6 | 2,557 | 791 | 316 | Liver biopsy | No | 8 |
| Stefano Ciardullo *et al* | USA | Cross-sectional | 2005-2016 | 2020 | 51.2 | 51.8 | 11,489 | 3,935 | 810 | Index | No | 8 |
| Yusuf Yilmaz *et al* | Turkey | Cohort | 2009-2018 | 2019 | 47.9 | 47 | 468 | 468 | 157 | Liver biopsy | No | 8 |
| Jie Han *et al* | China | Cross-sectional | 2014 | 2019 | 26.8 | 51.67 | 6,849 | 2,347 | 843 | Hepatic ultrasonography | No | 8 |
| Mahmud Mahamid *et al* | Israel | Case-control | 2010-2018 | 2019 | 64.5 | 63.7 | 995 | 242 | 91 | Mixed diagnostic methods | No | 7 |
| Xianghai Zhou *et al* | China | Cohort | 2012-2013 | 2019 | 39.1 | 49.65 | 3,166 | 716 | 116 | CT | No | 7 |
| Jing Wu *et al* | China | Cross-sectional | 2013-2014 | 2019 | 47.6 | 53.2 | 5,436 | 1,998 | 320 | Hepatic ultrasonography | No | 6 |
| Samer Gawrieh *et al* | USA | Cohort | 2004-2009 | 2019 | 26.8 | 52.21 | 534 | 534 | 103 | Liver biopsy | No | 8 |
| Diana Barb *et al* | USA | Cross-sectional | .. | 2019 | 73.8 | 50 | 187 | 94 | 51 | Liver biopsy | No | 6 |
| Xiuying Zhang *et al* | China | Cohort | 2013-2014 | 2019 | 39.4 | 50.11 | 3,122 | 708 | 225 | CT | No | 6 |
| Kexue Luo *et al* | China | Cross-sectional | 2015-2016 | 2019 | 41.2 | 68.5 | 515 | 515 | 96 | Hepatic ultrasonography | No | 6 |
| Tracey G. Simon *et al* | USA | Cross-sectional | 2000-2002 | 2019 | 43.4 | 63 | 3,876 | 688 | 148 | CT | No | 7 |
| YangFan Li *et al* | China | Case-control | 2015-2017 | 2019 | 68.3 | 48 | 1,086 | 543 | 26 | Hepatic ultrasonography | No | 7 |
| Sheng Tu *et al* | China | Cross-sectional | 2010-2016 | 2019 | 70.3 | 62.4 | 1,155 | 34 | 12 | Hepatic ultrasonography | No | 7 |
| Kristina Önnerhag *et al* | Sweden | Cohort | 1978-2006 | 2019 | 70.2 | 52.5 | 120 | 105 | 23 | Liver biopsy | No | 7 |
| Galit Weinstein *et al* | USA | Cross-sectional | 2011-2014 | 2019 | 48.0 | 61 | 1,287 | 378 | 77 | CT | No | 7 |
| Celal Ulasoglu *et al* | Turkey | Cohort | .. | 2019 | 53.6 | 48 | 515 | 515 | 205 | Liver biopsy | No | 7 |
| Hui Zhao *et al* | China | Cohort | 2013-2017 | 2019 | 53.3 | 38.33 | 1,165 | 363 | 35 | Hepatic ultrasonography | No | 7 |
| Toshifumi Tada *et al* | USA | Cohort | 2006-2016 | 2019 | 56.7 | 54 | 1,562 | 1,562 | 485 | Hepatic ultrasonography | No | 7 |
| Madunil Anuk Niriella *et al* | Sri Lanka | Cohort | 2007-2014 | 2019 | 45.7 | 54.2 | 2,985 | 936 | 311 | Hepatic ultrasonography | No | 7 |
| Hai-Yan Rong *et al* | China | Cross-sectional | 2017 | 2019 | 55.4 | 36.84 | 725 | 204 | 28 | Hepatic ultrasonography | No | 6 |
| Thierry Poynard *et al* | UK | Case-control | .. | 2019 | 63.7 | 58 | 869 | 505 | 136 | Liver biopsy | No | 6 |
| Amy E. McGhee-Jez *et al* | USA | Cohort | 2007-2017 | 2020 | 56.0 | 62 | 421 | 60 | 18 | Mixed diagnostic methods | No | 6 |
| Faryal Tahir *et al* | Pakistan | Cross-sectional | 2019 | 2020 | 51.0 | 36.93 | 96 | 96 | 26 | Hepatic ultrasonography | No | 7 |
| Hua Bian *et al* | China | Cross-sectional | .. | 2020 | 47.1 | 44 | 221 | 221 | 154 | Liver biopsy | No | 7 |
| Geraldine F. Clough *et al* | UK | Cross-sectional | .. | 2020 | 40.2 | 50.9 | 189 | 189 | 65 | Mixed diagnostic methods | No | 7 |
| Tomomi Kogiso *et al* | Japan | Cohort | 1990-2019 | 2020 | 50.6 | 53 | 544 | 544 | 272 | Liver biopsy | No | 7 |
| Jung Nam An *et al* | Korea | Cohort | 2013-2018 | 2020 | 48.6 | 53 | 455 | 455 | 168 | Liver biopsy | No | 7 |
| Quelson Coelho Lisboa *et al* | Brazil | Cross-sectional | .. | 2020 | 27.0 | 56 | 285 | 148 | 69 | Hepatic ultrasonography | No | 8 |
| James B Maurice *et al* | Mixed countries | Cross-sectional | .. | 2020 | 93.2 | 47.7 | 116 | 63 | 18 | Liver biopsy | No | 7 |
| Zeng-Pei Qiao *et al* | China | Cross-sectional | 2016-2019 | 2020 | 73.5 | 41.55 | 351 | 351 | 114 | Liver biopsy | No | 8 |
| Christian S. Alvarez *et al* | USA | Cohort | 1988-1994 | 2020 | 44.4 | 43 | 12,253 | 4,355 | 991 | Hepatic ultrasonography | No | 8 |
| Shunji Hirose *et al* | Japan | Cohort | 1975-2012 | 2020 | 66.4 | 43.27 | 223 | 223 | 48 | Liver biopsy | No | 6 |
| Hiroshi Ishiba *et al* | Japan | Cross-sectional | 2002-2015 | 2020 | 51.8 | 51 | 874 | 874 | 377 | Liver biopsy | No | 6 |
| Wei Xia *et al* | China | Case-control | 2015-2018 | 2020 | 56.0 | 70.24 | 325 | 111 | 73 | Hepatic ultrasonography | No | 7 |
| Paul R. Afolabi *et al* | UK | Cross-sectional | .. | 2020 | 60.8 | 51 | 97 | 97 | 36 | Liver biopsy | No | 7 |
| Chifa Ma *et al* | China | Cross-sectional | 2014-2015 | 2020 | 35.5 | 53.5 | 282 | 86 | 30 | Hepatic ultrasonography | No | 8 |
| Janus P. Ong *et al* | USA | Cohort | 1988-1994 | 2008 | 46.6 | .. | 12,822 | 817 | 100 | Index | No | 8 |
| HeidiS. Ahmed *et al* | USA | Cohort | 2000-2016 | 2020 | 38.1 | 47.61 | 700 | 700 | 223 | Mixed diagnostic methods | No | 8 |
| L. Giraldi *et al* | Italy | Case-control | 2005-2017 | 2020 | 62.5 | 51.37 | 815 | 371 | 40 | Mixed diagnostic methods | No | 7 |
| Jennifer Linge *et al* | UK | Cross-sectional | 2006-2010 | 2020 | .. | 62.65 | 9,545 | 1,204 | 159 | MRI | No | 6 |
| Chung-Hao Li *et al* | China | Cross-sectional | 2001-2010 | 2020 | 64.0 | 46.81 | 8,571 | 3,072 | 415 | Hepatic ultrasonography | No | 7 |
| Yinxia Su *et al* | China | Case-control | 2012-2014 | 2020 | 62.6 | 50.26 | 991 | 467 | 346 | Hepatic ultrasonography | No | 8 |
| Dan-Qin Sun *et al* | USA | Cross-sectional | 1988-1994 | 2020 | 46.9 | 43.63 | 12,571 | 4,552 | 890 | Hepatic ultrasonography | No | 7 |
| Alexandria Miller *et al* | USA | Cohort | 2014-2018 | 2020 | 58.6 | 70 | 181 | 49 | 30 | Mixed diagnostic methods | No | 7 |
| Dong Hyun Sinn *et al* | Korea | Cohort | 2003-2013 | 2020 | 51.2 | 52 | 111,492 | 37,263 | 6,151 | Hepatic ultrasonography | No | 8 |
| Su Lin *et al* | USA | Cross-sectional | 1988-1994 | 2020 | 46.8 | 43.89 | 12,045 | 4,347 | 1,092 | Hepatic ultrasonography | No | 7 |
| M Masudur Rahman *et al* | Bangladesh | Cross-sectional | 2014-2016 | 2020 | 30.4 | 46.1 | 1,305 | 242 | 82 | Hepatic ultrasonography | No | 7 |
| Xiaohan Wang *et al* | China | Cross-sectional | 2016 | 2021 | 48.0 | 59.84 | 342 | 105 | 26 | Index | No | 6 |
| Soichi Iritani *et al* | Japan | Cohort | 1976-2019 | 2020 | 60.1 | 52 | 446 | 446 | 147 | Mixed diagnostic methods | No | 6 |
| José Tadeu Stefano *et al* | Brazil | Cross-sectional | .. | 2020 | 38.2 | 55.7 | 102 | 102 | 70 | Liver biopsy | No | 6 |
| Fasiha Kanwal *et al* | USA | Cohort | 2004-2015 | 2020 | 94.3 | 54.52 | 271,906 | 271,906 | 78,065 | Mixed diagnostic methods | No | 8 |
| Karl Björkström *et al* | Sweden | Cohort | 1987-2016 | 2022 | 55.8 | 53.11 | 79,349 | 8,415 | 1,607 | Hospital record | No | 7 |
| Salih Boga *et al* | Germany | Cross-sectional | 2018-2019 | 2020 | 63.9 | 45.5 | 797 | 97 | 35 | Liver biopsy | No | 7 |
| Luca Miele *et al* | Italy | Case-control | 2010-2017 | 2022 | 47.7 | 57.15 | 918,954 | 151,431 | 37,413 | Hospital record | No | 7 |
| Cheng Han Ng *et al* | USA | Cross-sectional | 1999-2018 | 2022 | NA | 62 | 32,234 | 13,112 | 3,315 | Index | No | 7 |
| Therese Adrian *et al* | Denmark | Cross-sectional | 2015-2017 | 2021 | 61.5 | 64 | 741 | 291 | 51 | CT | No | 6 |
| Wen Dai *et al* | China | Cohort | 2011-2014 | 2021 | 45.8 | 51 | 16,093 | 1,717 | 204 | Hepatic ultrasonography | No | 7 |
| Ping-Fang Hu *et al* | China | Cross-sectional | 2016-2017 | 2021 | 42.4 | 41.9 | 3,717 | 1,217 | 208 | Hepatic ultrasonography | No | 8 |
| Khalid Alswat *et al* | Saudi Arabia | Cohort | 2009-2019 | 2021 | 40.5 | 42.6 | 832 | 832 | 294 | Liver biopsy | No | 7 |
| Nardeen Eldafashi *et al* | Mixed countries | Case-control | UK Newcastle:2004-2019 Berne:2010; Milan:2010-2016 | 2021 | 66.0 | 59.17 | 985 | 614 | 352 | Mixed diagnostic methods | No | 7 |
| Fátima Higuera‑de‑la‑Tijera *et al* | Mexico | Cross-sectional | 2010-2019 | 2021 | 26.0 | 46 | 222 | 222 | 75 | Liver biopsy | No | 7 |
| Peter C. Johnson *et al* | USA | Cross-sectional | 2011-2016 | 2021 | 60.6 | 48.77 | 33 | 33 | 12 | Liver biopsy | No | 6 |
| Sanjaya K. Satapathy *et al* | USA | Cohort | 2006-2015 | 2021 | 50.0 | 57 | 66 | 66 | 31 | Liver biopsy | No | 8 |
| Rui-Xu Yang *et al* | China | Cross-sectional | 2016-2018 | 2021 | 72.0 | 39 | 246 | 246 | 66 | Mixed diagnostic methods | No | 8 |
| Li-Wei Chen *et al* | China | Cross-sectional | 2013-2018 | 2021 | 32.4 | 56.8 | 2,713 | 1,566 | 320 | Hepatic ultrasonography | No | 8 |
| Hideki Fujii *et al* | Japan | Cross-sectional | .. | 2021 | 58.0 | 53 | 2,254 | 618 | 132 | Hepatic ultrasonography | No | 7 |
| Quentin M. Anstee *et al* | Mixed countries | Cross-sectional | 2018 | 2021 | 61.3 | 56 | 2,267 | 2,267 | 1263 | Hepatic ultrasonography | No | 7 |
| Basit Siddiqui *et al* | Pakistan | Cross-sectional | 2016-2018 | 2021 | 52.4 | 51.5 | 399 | 153 | 46 | Hepatic ultrasonography | No | 8 |
| Vincent J. H. Yao *et al* | USA | Cohort | .. | 2021 | 52.9 | 56 | 652 | 652 | 33 | Hepatic ultrasonography | No | 6 |
| Joon Ho Moon *et al* | Korea | Cohort | 2008-2015 | 2021 | 48.2 | 50.6 | 28,060 | 6,488 | 1,739 | Index | No | 6 |
| Eyun Song *et al* | Korea | Cross-sectional | 2016-2018 | 2021 | 54.2 | 46 | 5,661 | 1,393 | 162 | Mixed diagnostic methods | No | 7 |
| Jesús Funuyet-Salas *et al* | Spain | Cross-sectional | 2018 | 2021 | 62.5 | 52.6 | 307 | 307 | 57 | Liver biopsy | No | 6 |
| Mindie H. Nguyen *et al* | USA | Cohort | 2006-2013 | 2018 | 40.0 | .. | 91,558 | 91,558 | 34,384 | Hospital record | No | 6 |
| Zhengyu Hu *et al* | USA | Cross-sectional | 2005-2012 | 2021 | 51.4 | 42.3 | 2,436 | 698 | 149 | Index | No | 7 |
| Fuxi Li *et al* | China | Cross-sectional | 2009-2019 | 2021 | 77.9 | 45.6 | 4,010 | 1,790 | 557 | Hepatic ultrasonography | No | 6 |
| Anusha Vittal *et al* | USA | Cohort | 2016-2018 | 2021 | 36.0 | 54.2 | 93 | 61 | 24 | Hepatic ultrasonography | No | 7 |
| Jacqueline Bayliss *et al* | Australia | Cross-sectional | .. | 2021 | 25.0 | 45 | 152 | 107 | 28 | Liver biopsy | No | 7 |
| Stuart K Roberts *et al* | Australia | Cross-sectional | 2016-2018 | 2021 | 44.4 | 59.1 | 705 | 274 | 53 | Index | No | 7 |
| Masoud Baikpour *et al* | USA | Cross-sectional | 2014-2018 | 2021 | 44.0 | 50.3 | 123 | 123 | 35 | Mixed diagnostic methods | No | 7 |
| L. Gerber *et al* | USA | Cross-sectional | 2003-2006 | 2012 | 65.8 | 43.19 | 3,056 | 1,263 | 146 | Index | No | 7 |
| Leon A. Adams *et al* | Australia | Cohort | 1994-1995 | 2009 | 68.4 | 48.86 | 358 | 106 | 20 | Index | No | 6 |
| Evelyn Nunes Goulart da Silva Pereira *et al* | Brazil | Cross-sectional | 2008-2010 | 2021 | 56.0 | 50.81 | 305 | 305 | 72 | Hepatic ultrasonography | No | 7 |
| Hadi Emamat *et al* | Iran | Case-control | .. | 2021 | 43.1 | 42.3 | 999 | 196 | 33 | Mixed diagnostic methods | No | 7 |
| Katharine M. Irvine *et al* | Australia | Cohort | 2015-2017 | 2021 | 54.4 | 56.7 | 228 | 228 | 185 | Hepatic ultrasonography | No | 7 |
| Johnathan Huey Ming Lum *et al* | Singapore | Cohort | 2004-2017 | 2021 | 52.5 | 50.4 | 263 | 263 | 129 | Liver biopsy | No | 7 |
| Ke Zhu *et al* | China | Cross-sectional | 2018-2020 | 2021 | 57.4 | 47.9 | 1,677 | 365 | 32 | Hepatic ultrasonography | No | 8 |
| Junfeng Zhang *et al* | China | Cross-sectional | 2011 | 2021 | 29.9 | 53.41 | 1,318 | 781 | 267 | Index | No | 7 |
| Isabel Belinchón‑Romero *et al* | Spain | Cohort | 2017-2018 | 2021 | 55.8 | 53 | 215 | 91 | 23 | Hepatic ultrasonography | No | 6 |
| Christian Labenz *et al* | Germany | Case-control | 2000-2015 | 2021 | 52.3 | 60.3 | 57,483 | 57,483 | 27,937 | Hepatic ultrasonography | No | 7 |
| Rui Huang *et al* | China | Cross-sectional | 2019 | 2021 | 65.8 | 43.8 | 5,181 | 5,181 | 801 | Liver biopsy | No | 7 |
| Yasir Mohammed Khayyat *et al* | Saudi Arabia | Cross-sectional | 2016-2019 | 2021 | 55.4 | 52.33 | 1,262 | 1,262 | 626 | Hepatic ultrasonography | No | 7 |
| Diego García-Compeán *et al* | USA | Cross-sectional | 2015 | 2020 | 29.8 | 47.8 | 695 | 695 | 110 | Index | No | 8 |
| Chao Sang *et al* | China | Cohort | .. | 2021 | 52.2 | 46.76 | 540 | 540 | 370 | Liver biopsy | No | 7 |
| Yariv Gerber *et al* | USA | Cohort | 1985-1986 | 2021 | 43.0 | 50.1 | 2,809 | 673 | 204 | CT | No | 8 |
| Julia Blomdahl *et al* | Sweden | Cross-sectional | 1988-2018 | 2021 | 70.9 | 59.8 | 86 | 86 | 42 | Liver biopsy | No | 8 |
| Xiaotao Zhang *et al* | USA | Cross-sectional | 2017-2018 | 2021 | 48.2 | 48.4 | 4,024 | 2,373 | 647 | Hepatic ultrasonography | No | 6 |
| Tracey G. Simon *et al* | Sweden | Cohort | 1966-2016 | 2021 | 56.7 | 49.9 | 39,907 | 8,892 | 687 | Liver biopsy | No | 7 |
| Guyi Wang *et al* | China | Cohort | 2020 | 2021 | 50.5 | 46 | 218 | 86 | 9 | Hepatic ultrasonography | Yes | 6 |
| Bin Wang *et al* | China | Cohort | 2010 | 2021 | 30.8 | 57.9 | 8,451 | 2,557 | 879 | Hepatic ultrasonography | No | 7 |
| Karlijn J. Nass *et al* | Netherlands | Cohort | 2006-2013 | 2017 | 38.0 | 43.63 | 22,865 | 4,790 | 205 | Index | No | 7 |
| Zobair M. Younossi *et al* | USA | Cross-sectional | 2001-2020 | 2021 | 43.8 | 53.1 | 829 | 829 | 294 | Liver biopsy | No | 7 |
| Luis A. Rodriguez *et al* | USA | Cross-sectional | 2000-2002 | 2021 | 46.9 | 62 | 6,195 | 385 | 88 | CT | No | 7 |
| Francesco Baratta *et al* | Italy | Cohort | .. | 2021 | 60.2 | 56.23 | 987 | 795 | 231 | Mixed diagnostic methods | No | 8 |
| Laurens van Kleef *et al* | Netherlands | Cross-sectional | 2009-2014 | 2021 | 41.5 | 68.45 | 5,445 | 1,566 | 416 | Hepatic ultrasonography | No | 8 |
| Qi Huang *et al* | USA | Cohort | 1988-1994 | 2021 | 48.7 | 42.1 | 12,480 | 3,779 | 895 | Hepatic ultrasonography | No | 7 |
| Weiti Wu *et al* | USA | Cross-sectional | 1988-1994 | 2021 | 44.1 | 57.72 | 4,037 | 4,037 | 483 | Hepatic ultrasonography | No | 7 |
| Ramy Younes *et al* | Mixed countries | Cohort | 1990-2016 | 2021 | 64.5 | 48 | 1,339 | 1,339 | 377 | Liver biopsy | No | 8 |
| Nasim Aslam Ghumman *et al* | Pakistan | Cross-sectional | .. | 2021 | 52.5 | 40.4 | 80 | 80 | 21 | Hepatic ultrasonography | No | 6 |
| Donghee Kim *et al* | USA | Cohort | 1988-1994 | 2021 | 49.7 | 45.2 | 7,761 | 2,438 | 327 | Hepatic ultrasonography | No | 8 |
| Huiyul Park *et al* | Korea | Cohort | .. | 2021 | .. | .. | 6,775 | 2,385 | 303 | Hepatic ultrasonography | No | 8 |
| Khalid A. Alsuhaibani *et al* | Saudi Arabia | Cross-sectional | 2016 | 2021 | 45.0 | 50.3 | 346 | 346 | 68 | Hepatic ultrasonography | No | 7 |
| Anastasia-Stefania Alexopoulos *et al* | USA | Cohort | 2007-2019 | 2021 | 41.5 | 53 | 713 | 713 | 348 | Liver biopsy | No | 8 |
| Kanokwan Pinyopornpanish *et al* | USA | Cross-sectional | 2015-2020 | 2021 | 41.4 | .. | 392,800 | 392,800 | 171,570 | Liver biopsy | No | 6 |
| Magnus Holmer *et al* | Sweden | Cohort | 1974-2019 | 2022 | 62.0 | 51 | 546 | 100 | 19 | Liver biopsy | No | 8 |
| Ayelet Grupper *et al* | Israel | Cohort | 2015-2021 | 2022 | 66.3 | 57.4 | 341 | 124 | 67 | Hepatic ultrasonography | No | 7 |
| Pegah Golabi *et al* | USA | Cohort | 1988-1994 | 2022 | 56.2 | 43.5 | 3,981 | 3,981 | 596 | Mixed diagnostic methods | No | 9 |
| Anshuman Elhence *et al* | India | Cohort | 2014-2021 | 2022 | 52.4 | 39.91 | 330 | 330 | 102 | Liver biopsy | No | 7 |
| Sofi Damjanovska *et al* | USA | Cross-sectional | 2010-2019 | 2022 | 47.0 | 52 | 93 | 93 | 48 | Mixed diagnostic methods | No | 6 |
| Michelle Ferrari-Cestari *et al* | UK | Cohort | 2015-2017 | 2022 | 49.6 | 56.7 | 250 | 228 | 185 | Hepatic ultrasonography | No | 7 |
| Aaron P. Thrift *et al* | USA | Cross-sectional | .. | 2022 | 44.6 | 53.8 | 202 | 82 | 32 | Hepatic ultrasonography | No | 9 |
| Shivaram P Singh *et al* | India | Cross-sectional | .. | 2022 | 81.7 | 42.3 | 633 | 633 | 209 | Hepatic ultrasonography | No | 6 |
| Shivaram P Singh *et al* | USA | Cross-sectional | .. | 2022 | 56.3 | 49.7 | 451 | 451 | 190 | Hepatic ultrasonography | No | 6 |
| Jiaxuan Wang *et al* | China | Cross-sectional | 2016-2021 | 2022 | 75.1 | 50.92 | 4,335 | 4,335 | 772 | Hepatic ultrasonography | No | 7 |
| Yuya Seko *et al* | Japan | Cohort | 1994-2020 | 2022 | 42.9 | 57 | 1,395 | 1,395 | 505 | Liver biopsy | No | 6 |
| Mehmet Sayiner *et al* | USA | Cross-sectional | 2006-2016 | 2020 | 45.4 | 70.11 | 1,980,950 | 1,980,950 | 1,709,336 | Hospital record | No | 7 |
| Valentin Blank *et al* | Germany | Cohort | .. | 2022 | 49.1 | 57.1 | 57 | 57 | 34 | Hepatic ultrasonography | No | 7 |
| Chandrashekaraiah Bharath Kumar *et al* | India | Case-control | 2013-2014 | 2022 | 75.7 | 55 | 177 | 118 | 61 | Liver biopsy | No | 6 |
| Srinevas K. Reddy *et al* | USA | Cross-sectional | .. | 2013 | 56.6 | 52.3 | 303,396 | 32,347 | 14,027 | Hospital record | No | 7 |
| Albrecht Boehlig *et al* | Germany | Cohort | 2019-2020 | 2022 | 47.1 | 55.8 | 137 | 121 | 40 | Hepatic ultrasonography | No | 8 |
| Samuel O. Antwi *et al* | USA | Case-control | .. | 2022 | 34.5 | 76.79 | 11,145 | 11,145 | 7,624 | Liver biopsy | No | 6 |
| Thaís Grecca Andrade *et al* | Brazil | Cross-sectional | 2000-2018 | 2022 | 33.6 | 48 | 143 | 137 | 61 | Liver biopsy | No | 7 |
| Yingying Chen *et al* | China | Cross-sectional | 2011-2020 | 2022 | 53.2 | 47 | 67,616 | 15,937 | 2,412 | Mixed diagnostic methods | No | 7 |
| Olivier Deckmyn *et al* | USA | Cohort | 2018-2020 | 2022 | 54.1 | 54.19 | 449,329 | 428,774 | 89,522 | Liver biopsy | No | 6 |
| Hyoeun Kim *et al* | Korea | Cross-sectional | 2016-2019 | 2022 | 63.4 | 56.5 | 2,144 | 891 | 188 | Hepatic ultrasonography | No | 7 |
| Emily Truong *et al* | USA | Cross-sectional | 2017-2018 | 2022 | .. | 49.3 | 3,639 | 3,639 | 836 | Hepatic ultrasonography | No | 7 |
| Qingling Wang *et al* | China | Cross-sectional | 2018-2021 | 2022 | 66.2 | 41 | 136 | 136 | 59 | Liver biopsy | No | 7 |
| Anca Trifan *et al* | Romania | Cohort | 2019-2021 | 2022 | 56.8 | 56 | 331 | 331 | 101 | Hepatic ultrasonography | No | 9 |
| Meagan E. Gray *et al* | USA | Cross-sectional | 2010-2011 | 2022 | 42.2 | 50.3 | 1,726 | 408 | 110 | CT | No | 8 |
| Zobair M. Younossi *et al* | Mixed countries | Cohort | 2017-2019 | 2022 | 40.4 | 58 | 1,679 | 1,679 | 1,237 | Liver biopsy | No | 8 |
| Taoping Sun *et al* | China | Case-control | 2013 | 2022 | 65.0 | 54.8 | 1,880 | 940 | 252 | Hepatic ultrasonography | No | 7 |
| Carlo Saitta *et al* | Italy | Cross-sectional | 2018-2019 | 2022 | 59.6 | 55.4 | 374 | 374 | 135 | Hepatic ultrasonography | No | 7 |
| Mary E. Rinella *et al* | USA | Cohort | 1997-2017 | 2022 | 52.0 | 59.3 | 938 | 938 | 572 | Mixed diagnostic methods | No | 8 |
| Vera Karamfifilova *et al* | Bulgaria | Cross-sectional | .. | 2022 | 9.0 | 50.8 | 77 | 77 | 30 | Hepatic ultrasonography | No | 6 |
| Pimsiri Sripongpun *et al* | USA | Cohort | 2012-2017 | 2022 | 38.5 | 49 | 676 | 676 | 155 | Liver biopsy | No | 6 |
| Kristina-Ana Klaric *et al* | USA | Cross-sectional | 2016-2017 | 2022 | 61.4 | 51 | 376 | 86 | 34 | Liver biopsy | No | 7 |
| Takanori Ito *et al* | Mixed countries | Cross-sectional | 1995-2019 | 2022 | 54.1 | 46 | 1,489 | 1,489 | 454 | Liver biopsy | No | 7 |
| Alexa M. Giammarino *et al* | USA | Cohort | 2015-2020 | 2022 | 34.9 | 51.89 | 614 | 614 | 181 | Liver biopsy | No | 8 |
| Cheng Yu *et al* | China | Cohort | 2011-2013 | 2022 | 63.5 | 45.62 | 30,633 | 5,769 | 768 | Hepatic ultrasonography | No | 7 |
| Shirley X Jiang *et al* | Canada | Cross-sectional | 2015-2018 | 2022 | 50.0 | 51.1 | 224 | 164 | 99 | Hepatic ultrasonography | No | 8 |
| Alexandre Pariente *et al* | France | Cohort | 2020 | 2022 | 56.8 | 56.9 | 671 | 671 | 327 | Mixed diagnostic methods | No | 7 |
| Anna Kotronen *et al* | Finland | Cohort | 2007 | 2010 | .. | 59.5 | 2,766 | 581 | 145 | Index | No | 8 |
| Paulina Moctezuma‑Velázquez *et al* | Mexico | Cohort | 2002 | 2022 | 83.0 | 51 | 470 | 359 | 111 | CT | Yes | 7 |
| Abdel-Rauf Zeina *et al* | Israel | Cohort | 2010-2019 | 2022 | 41.6 | 64.53 | 377 | 101 | 41 | Hepatic ultrasonography | No | 6 |
| Eline H. van den Berg *et al* | Netherlands | Cohort | 2006-2013 | 2017 | 37.9 | 44 | 37,496 | 37,496 | 1,186 | Index | No | 7 |
| Jianqi Zhao *et al* | China | Cross-sectional | .. | 2022 | 62.7 | 57.1 | 424 | 169 | 24 | Hepatic ultrasonography | No | 7 |
| G Masterton *et al* | UK | Cohort | 2006-2009 | 2010 | 49.0 | 55.8 | 143 | 143 | 78 | Hospital record | No | 6 |

CT, computed tomography; MRI, magnetic resonance imaging; NAFLD, non-alcoholic fatty liver disease.

**Table S2. Characteristics of studies reporting the prevalence of type 2 diabetes in patients with NAFLD: source of heterogeneity**

|  | Studies, n | Median | Mean | Range |
| --- | --- | --- | --- | --- |
| Mean age, years | 334 | 50.6 | 51.1 | 33.1-78.0 |
| Male, % | 329 | 51.2 | 51.2 | 9.0-94.3 |
| Publication year | 340 | 2019 | 2017 | 2002-2022 |
|  | Studies, n | Total patients of type 2 diabetes  (inter-study range) | | Prevalence range |
| Diagnose method |  |  | |  |
| *Index* | 20 | 146 (89-559) | | 19.8 (13.8-25.1) |
| *Hepatic ultrasonography* | 131 | 117 (36-357) | | 23.5 (13.4-34.6) |
| *MRI* | 3 | 42 (24-101) | | 13.2 (12.7-29.9) |
| *CT* | 12 | 99 (63-124) | | 22.2 (17.2-27.8) |
| *Liver biopsy* | 112 | 117 (36-357) | | 23.6 (13.4-34.8) |
| *Hospital record* | 19 | 24,381 (3,288-71,673) | | 48.1 (32.0-58.1) |
| *Multiple diagnostic methods* | 43 | 79 (42-155) | | 28.7 (17.2-40.7) |
| Region |  |  | |  |
| *East Asia* | 102 | 152 (62-367) | | 25.7 (13.7-35.9) |
| *South Asia* | 16 | 45 (25-106) | | 32.0 (20.1-50.0) |
| *Southeast Asia* | 7 | 66 (40-111) | | 23.5 (14.5-43.5) |
| *West Asia* | 27 | 46 (21-80) | | 28.6 (17.6-36.5) |
| *North America* | 92 | 147 (49-609) | | 29.5 (22.6-40.5) |
| *South America* | 12 | 71 (62-87) | | 39.1 (28.2-52.9) |
| *Australia* | 7 | 53 (34-121) | | 25.9 (21.2-58.1) |
| *Europe* | 65 | 69 (37-185) | | 26.9 (16.6-38.8) |
| *Africa* | 4 | 45 (40-49) | | 46.3 (42.1-51.3) |
| *Mixed ^a^* | 8 | 365 (228-729) | | 47.1 (29.2-56.1) |

CT, computed tomography; MRI, magnetic resonance imaging; NAFLD, non-alcoholic fatty liver disease.

^a^ Mixed diagnostic methods refer to the definition of NAFLD in a study using more than one diagnostic method.

**Table S3. Average annual percent change (%) in the prevalence of type 2 diabetes among NAFLD populations globally and by region**

|  | Years reported | AAPC (95% CI) | P value |
| --- | --- | --- | --- |
| Global | 1966-2020 | 2.9 (1.3 to 4.5) | <0.001 |
| By region |  |  |  |
| East Asia | 1966-2018 | 1.6 (-1.6 to 4.8) | 0.30 |
| South Asia | 2000-2016 | 1.9 (-33.4 to 56.1) | 0.90 |
| Southeast Asia | 1988-2014 | 4.1 (1.7 to 6.7) | <0.001 |
| West Asia | 1988-2020 | -2.2 (-22.7 to 23.6) | 0.90 |
| North America | 1974-2019 | 0.3 (-16.0 to 19.7) | 0.99 |
| South America | 1987-2019 | 5.1 (2.5 to 7.7) | <0.001 |
| Australia | 1994-2014 | 10.7 (6.8 to 14.6) | <0.001 |
| Europe | 1990-2018 | -2.3 (-14.8 to 12.0) | 0.70 |
| Africa | 2003-2005 | .. | .. |

AAPC, average annual percent change; CI, confidence interval; NAFLD, non-alcoholic fatty liver disease.

**Table S4. The prevalence of type 2 diabetes among patients with NAFLD-stratified by age, region, publication year, sample size and diagnosis of NAFLD (excluding index and hospital record of NAFLD)**

|  | Studies, n | Prevalence % (95% CI) | I2 |
| --- | --- | --- | --- |
| Global | 299 | 28.2 (26.4-30.1) | 100.0% |
| Study design |  |  |  |
| *Cross-sectional* | 166 | 26.6 (23.4-30.0) | 100.0% |
| *Cohort* | 108 | 28.6 (26.2-31.1) | 100.0% |
| *Case-control* | 25 | 38.4 (31.6-45.8) | 100.0% |
| Average age |  |  |  |
| *<50.3 years* | 147 | 23.4 (21.2-25.7) | 99.9% |
| *≥50.3 years* | 148 | 33.7 (31.6-35.9) | 99.9% |
| *NA* | 4 | 22.7 (9.3-45.8) | 100.0% |
| Region |  |  |  |
| *East Asia* | 93 | 23.4 (20.8-26.2) | 99.9% |
| *South Asia* | 16 | 31.0 (25.6-37.0) | 98.7% |
| *Southeast Asia* | 7 | 27.4 (13.4-47.7) | 99.8% |
| *West Asia* | 26 | 26.1 (17.7-36.8) | 99.8% |
| *North America* | 74 | 29.2 (26.4-32.2) | 100.0% |
| *South America* | 12 | 41.0 (31.7-50.9) | 99.1% |
| *Australia* | 5 | 51.6 (23.9-78.3) | 99.7% |
| *Europe* | 55 | 28.2 (22.4-34.7) | 99.9% |
| *Africa* | 2 | 51.7 (41.1-62.1) | 92.4% |
| *Mixed* | 8 | 45.7 (32.8-59.2) | 99.8% |
| Publication-year |  |  |  |
| *<2018* | 128 | 25.3 (22.7-28.1) | 99.9% |
| *≥2018* | 171 | 30.5 (28.4-32.7) | 100.0% |
| Sample size |  |  |  |
| *<468 participants* | 149 | 34.7 (31.9-37.6) | 99.05 |
| *≥468 participants* | 150 | 22.6 (20.4-25.0) | 100.0% |
| Quality grade |  |  |  |
| *5* | 4 | 40.4 (25.9-56.9) | 97.1% |
| *6* | 85 | 27.1 (24.3-30.0) | 100.0% |
| *7* | 139 | 28.3 (22.8-34.4) | 100.0% |
| *8* | 767 | 29.4 (25.8-33.4) | 99.95 |
| *9* | 4 | 27.3 (16.3-41.9) | 99.3% |
| Diagnosis of NAFLD |  |  |  |
| *Hepatic ultrasonography* | 131 | 23.9 (20.9-27.2) | 100.0% |
| *MRI* | 3 | 20.9 (7.6-46.1) | 99.2% |
| *CT* | 12 | 22.2 (18.8-26.1) | 98.3% |
| *Liver biopsy* | 112 | 34.5 (30.8-38.4) | 99.9% |
| *Mixed ^a^* | 43 | 29.5 (26.0-33.3) | 99.8% |

CI, confidence interval; CT, computed tomography; MRI, magnetic resonance imaging; NAFLD, non-alcoholic fatty liver disease.

^a^ Mixed diagnostic methods refer to the definition of NAFLD in a study using more than one diagnostic method.

**Table S5. Secondary analysis of the prevalence of type 2 diabetes among patients with NAFLD**

|  | Studies, n | Prevalence % (95% CI) | *I^2^* |
| --- | --- | --- | --- |
| Sex |  |  |  |
| *Male* | 19 | 15.7 (11.7-20.7) | 99.8% |
| *Female* | 25 | 22.2 (17.2-28.0) | 99.8% |
| Obesity |  |  |  |
| *Non-lean* | 23 | 25.9 (20.3-32.5) | 99.8% |
| *Lean* | 19 | 15.2 (10.8-21.0) | 99.5% |
| Metabolically |  |  |  |
| *Healthy* | 4 | 6.9 (3.0-15.1) | 99.8% |
| *At-risk* | 4 | 26.8 (19.1-36.1) | 99.3% |
| Category |  |  |  |
| *NAFL* | 19 | 24.6 (17.0-34.1) | 99.9% |
| *NASH* | 22 | 40.4 (28.6-53.5) | 99.4% |
| *Hepatic fibrosis* | 31 | 44.2 (33.2-55.7) | 100.0% |
| *HCC* | 8 | 67.8 (55.5-78.1) | 99.4% |
| Severity |  |  |  |
| *Mild* | 8 | 17.5 (11.7-25.3) | 99.9% |
| *Moderate to severe* | 8 | 29.4 (20.3-40.5) | 99.8% |
| Covid-19 |  |  |  |
| *No* | 338 | 28.4 (25.2-31.7) | 100.0% |
| *Yes* | 2 | 18.7 (5.8-46.1) | 99.2% |

CI, confidence interval; HCC, hepatocellular carcinoma; NAFL, non-alcoholic fatty liver; NAFLD, non-alcoholic fatty liver disease; NASH, non-alcoholic steatohepatitis.

**Table S6. Univariable and multivariable meta-regression analyses on the prevalence of type 2 diabetes among patients with NAFLD**

|  | Studies, n | Univariable analysis | | | Multivariable analysis | |
| --- | --- | --- | --- | --- | --- | --- |
|  |  | OR (95% CI) | *P* value | R^2^ (%) | OR (95% CI) | *P* value |
| Study design | 340 |  | 0.02 | 1.63 |  | 0.20 |
| *Cross-sectional* | 185 | Ref. |  |  | Ref. |  |
| *Cohort* | 129 | 1.10 (0.90-1.34) | 0.35 |  | 0.95 (0.79-1.14) | 0.59 |
| *Case-control* | 26 | 1.67 (1.15-2.40) | 0.01 |  | 1.22 (0.88-1.69) | 0.24 |
| Region | 340 |  | 0.29 | 5.03 |  | 0.13 |
| *East Asia* | 102 | Ref. |  |  | Ref. |  |
| *West Asia* | 27 | 1.11 (0.76-1.61) | 0.59 |  | 1.17 (0.84-1.63) | 0.35 |
| *South Asia* | 16 | 1.42 (0.89-2.26) | 0.14 |  | 1.59 (1.05-2.39) | 0.03 |
| *Southeast Asia* | 7 | 1.19 (0.60-2.32) | 0.62 |  | 1.24 (0.68-2.25) | 0.48 |
| *North America* | 92 | 1.38 (1.08-1.77) | 0.01 |  | 1.07 (0.84-1.35) | 0.60 |
| *South America* | 12 | 2.19 (1.29-3.70) | 0.004 |  | 1.59 (0.98-2.56) | 0.06 |
| *Europe* | 65 | 1.12 (0.85-1.47) | 0.41 |  | 2.50 (1.16-5.41) | 0.26 |
| *Africa* | 4 | 2.81 (1.17-6.77) | 0.02 |  | 2.21 (1.22-3.98) | 0.02 |
| *Australia* | 7 | 2.18 (1.11-4.28) | 0.02 |  | 0.87 (0.69-1.11) | 0.01 |
| *Mixed* | 8 | 2.65 (1.41-4.98) | 0.003 |  | 1.64 (0.93-2.88) | 0.09 |
| Mean age | 340 | 1.005 (1.004-1.007) | <0.0001 | 11.31 | 1.01 (1.003-1.01) | <0.0001 |
| Male, % | 338 | 0.998 (0.997-0.999) | 0.03 | 1.07 | 0.999 (0.998-1.00) | 0.15 |
| Publication, year | 340 | 1.03 (1.01-1.05) | 0.01 | 1.62 | 1.02 (1.00-1.04) | 0.03 |
| Diagnosis of NAFLD | 340 |  | <0.0001 | 11.90 |  | <0.0001 |
| *Hepatic ultrasonography* | 138 | Ref. |  |  | Ref. |  |
| *Hospital record* | 19 | 2.65 (1.76-3.97) | <0.0001 |  | 2.04 (1.38-3.01) | <0.0001 |
| *Index* | 20 | 0.69 (0.47-1.01) | 0.06 |  | 0.67 (0.47-0.95) | 0.03 |
| *Liver biopsy* | 116 | 1.67 (1.35-2.07) | <0.0001 |  | 1.79 (1.45-2.21) | <0.0001 |
| *MRI* | 4 | 0.85 (0.32-2.24) | 0.74 |  | 0.88 (0.36-2.13) | 0.78 |
| *CT* | 12 | 0.91 (0.56-1.51) | 0.72 |  | 0.72 (0.45-1.14) | 0.16 |
| *Mixed ^a^* | 46 | 1.34 (1.00-1.80) | 0.05 |  | 1.39 (1.06-1.83) | 0.02 |
| Quality grade | 340 | 1.03 (0.91-1.16) | 0.68 | 0.25 | 1.04 (0.93-1.17) | 0.45 |

CI, confidence interval; CT, computed tomography; MRI, magnetic resonance imaging; NAFLD, non-alcoholic fatty liver disease; OR, odd ratio.

^a^ Mixed diagnostic methods refer to the definition of NAFLD in a study using more than one diagnostic method.

**Table S7. Characteristics of the included studies in the prevalence of type 2 diabetes among MAFLD patients**

| Author | Country | Study design | Study year | Publication year | Male (%) | Average age | Sample size | Population of MAFLD | Events | Quality grade |
| --- | --- | --- | --- | --- | --- | --- | --- | --- | --- | --- |
| Jiaofeng Huang *et al* | USA | Cross-sectional | 1988-1994 | 2021 | 49.8 | 48.39 | 13,083 | 4,087 | 1,171 | 6 |
| Hyoeun Kim *et al* | Korea | Cross-sectional | 2016-2019 | 2022 | 63.4 | 56.3 | 2,144 | 995 | 225 | 7 |
| Yunlei Den *et al* | USA | Cross-sectional | 2017-2018 | 2021 | 48.6 | 51.37 | 4,869 | 4,748 | 996 | 6 |
| Jiahua Fan *et al* | China | Cross-sectional | 2018-2019 | 2021 | 40.4 | 67 | 5,377 | 1,571 | 392 | 7 |
| Guan Huei Lee *et al* | Singapore | Case-control | .. | 2020 | 59.7 | 57 | 144 | 72 | 29 | 6 |
| Na Li *et al* | China | Case-control | 2019-2020 | 2021 | 33.9 | 35.1 | 62 | 30 | 17 | 7 |
| Hideki Fujii *et al* | Japan | Cross-sectional | .. | 2021 | 58.0 | 53 | 2,254 | 789 | 178 | 7 |
| Liang-Jie Tang *et al* | China | Cohort | 2017-2020 | 2022 | 67.0 | 43 | 851 | 851 | 322 | 8 |
| Masato Yoneda *et al* | Japan | Cohort | 2013-2019 | 2021 | 62.4 | 46.2 | 3,995,637 | 237,242 | 48,784 | 7 |
| Cheng Yu *et al* | China | Cohort | 2011-2013 | 2022 | 63.5 | 45.62 | 30,633 | 6,442 | 968 | 7 |
| Xiaomo Wang *et al* | China | Cohort | 2006-2012 | 2021 | 81.1 | 50.21 | 152,139 | 47,995 | 7,411 | 8 |
| Juan Carlos Rodriguez-Duque *et al* | Spain | Case-control | 2018-2019 | 2022 | 48.4 | 51.33 | 2,549 | 121 | 47 | 6 |
| Huiyul Park *et al* | Korea | Cross-sectional | 2017-2020 | 2021 | 80.6 | 47 | 6,775 | 3,021 | 401 | 7 |
| Dan-Qin Sun *et al* | USA | Cross-sectional | 1988-1994 | 2020 | 46.9 | 43.63 | 12,571 | 3,794 | 896 | 7 |
| Francesco Baratta *et al* | Italy | Cohort | .. | 2021 | 60.2 | 56.23 | 987 | 816 | 238 | 8 |
| Laurens van Kleef *et al* | Netherlands | Cross-sectional | 2009-2014 | 2021 | 41.5 | 68.42 | 5,445 | 300 | 53 | 8 |
| Su Lin *et al* | USA | Cross-sectional | 1988-1994 | 2020 | 46.8 | 43.65 | 13,083 | 3,885 | 1,171 | 7 |
| Qi Huang *et al* | USA | Cohort | 1988-1994 | 2021 | 48.7 | 47.4 | 12,480 | 3,909 | 1,085 | 7 |
| Georg Semmler *et al* | Australia | Cohort | 2007-2020 | 2021 | 53.0 | 60.4 | 4,718 | 2,189 | 569 | 8 |
| Sheng Yang *et al* | USA | Cross-sectional | 2017-2018 | 2021 | 46.8 | 59.9 | 1,186 | 595 | 207 | 7 |
| Donghee Kim *et al* | USA | Cohort | 1988-1994 | 2021 | 49.7 | 47.1 | 7,761 | 2,256 | 354 | 8 |
| Mimi Kim *et al* | Korea | Cross-sectional | 2017-2020 | 2022 | 80.6 | 47.4 | 6,775 | 3,021 | 401 | 8 |
| Rieko Bessho *et al* | Japan | Cross-sectional | 2012-2018 | 2022 | 67.2 | 60.2 | 890 | 384 | 84 | 8 |
| Giovanni Alejandro Salgado Alvarez *et al* | USA | Cross-sectional | 2020 | 2022 | 23.9 | 54.4 | 125 | 125 | 28 | 6 |
| Shuhei Fukunaga *et al* | Japan | Cohort | 2008-2021 | 2022 | 73.2 | 51 | 9,100 | 2,416 | 234 | 7 |
| Yanbo Guo *et al* | China | Cohort | 2016 | 2022 | 51.9 | 36.47 | 11,444 | 1,681 | 183 | 8 |
| Karolina Drożdż *et al* | Poland | Cross-sectional | .. | 2022 | 53.9 | 59.14 | 191 | 191 | 124 | 8 |
| Yasuhiro Matsubayashi *et al* | Japan | Cohort | 2008 | 2022 | 58.6 | 44.84 | 570,426 | 114,986 | 22,941 | 8 |
| R. Jamali *et al* | Iran | Cohort | 2017-2018 | 2022 | 54.0 | 51.64 | 200 | 200 | 163 | 7 |
| Dandan Peng *et al* | China | Cross-sectional | 2021-2022 | 2022 | 67.5 | 48.84 | 228 | 171 | 32 | 8 |
| Fang Lei *et al* | China | Cohort | 2009-2017 | 2022 | 57.9 | 44.96 | 2,083,984 | 701,718 | 89,073 | 8 |
| Vítor Macedo Silva *et al* | Portugal | Cohort | .. | 2022 | 47.0 | 53 | 117 | 117 | 42 | 5 |
| Weitao Su *et al* | China | Cross-sectional | 2020-2021 | 2022 | 36.5 | 58 | 1,878 | 1,878 | 733 | 7 |
| Ji Yeon Seo *et al* | Korea | Cohort | 2017-2019 | 2022 | 85.4 | 54.2 | 6,414 | 3,198 | 642 | 8 |
| Liang‑Jie Tang *et al* | China | Cohort | 2017-2020 | 2022 | 72.7 | 43.09 | 469 | 469 | 190 | 8 |
| Yuying Wang *et al* | China | Cross-sectional | .. | 2022 | 40.5 | 56.7 | 12,183 | 5,891 | 1,261 | 8 |
| Jialu Yang *et al* | China | Cross-sectional | 2018-2019 | 2022 | 40.1 | 63.57 | 5,011 | 1,423 | 348 | 7 |
| Ya‑Cong Zhang *et al* | USA | Cohort | 1988-1994 | 2022 | 46.2 | 42.68 | 11,000 | 3,407 | 748 | 8 |
| Xiaoning Chen *et al* | USA | Cross-sectional | 2017-2018 | 2022 | 52.9 | 50.7 | 2,622 | 2,622 | 706 | 7 |
| Xiaoning Chen *et al* | Mixed countries | Cross-sectional | .. | 2022 | 53.6 | 49.47 | 293 | 293 | 161 | 7 |

MAFLD, metabolic associated fatty liver disease.

**Table S8. Characteristics of studies reporting the prevalence of type 2 diabetes in patients with MAFLD: source of heterogeneity**

|  | Studies, n | Median | Mean | Range |
| --- | --- | --- | --- | --- |
| Mean age, years | 40 | 50.9 | 51.2 | 35.1-68.4 |
| Male, % | 40 | 53.3 | 55.4 | 23.9-85.4 |
| Publication year | 40 | 2022 | 2022 | 2020-2022 |
|  | Studies, n | Total patients of type 2 diabetes  (inter-study range) | | Range |
| Region |  |  | |  |
| *East Asia* | 21 | 401 (234-1,261) | | 20.6 (15.0-24.5) |
| *Southeast Asia* | 1 | .. | | .. |
| *West Asia* | 1 | .. | | .. |
| *East and southeast Asia* | 1 | .. | | .. |
| *North America* | 10 | 822 (442-1,063) | | 23.6 (21.0-28.7) |
| *Australia* | 1 | .. | | .. |
| *Europe* | 5 | 117 (53-191) | | 35.9 (29.2-38.8) |

MAFLD, metabolic associated fatty liver disease.

**Table S9. Average annual percent change (%) in the prevalence of type 2 diabetes among MAFLD populations globally and by region**

|  | Years reported | AAPC (95% CI) | P value |
| --- | --- | --- | --- |
| Global | 1988-2021 | 3.0 (1.0 to 5.0) | <0.001 |
| By region |  |  |  |
| East Asia | 2006-2021 | 8.2 (-18.3 to 32.3) | 0.60 |
| West Asia | 2017 | .. | .. |
| North America | 1988-2020 | 0.1 (-5.1 to 5.6) | 0.90 |
| Australia | 2007 | .. | .. |
| Europe | 2009-2018 | .. | .. |

AAPC, average annual percent change; CI, confidence interval; MAFLD, metabolic associated fatty liver disease.

**Table S10. Univariable and multivariable meta-regression analyses on the prevalence of type 2 diabetes among patients with MAFLD**

|  | Studies, n | Univariable analysis | | | Multivariable analysis | |
| --- | --- | --- | --- | --- | --- | --- |
|  |  | OR (95% CI) | *P* value | R^2^ (%) | OR (95% CI) | *P* value |
| Study design | 40 |  | 0.38 | 0.68 |  | 0.27 |
| *Cross-sectional* | 20 | Ref. |  |  | Ref. | 0.44 |
| *Cohort* | 17 | 0.89 (0.55-1.44) | 0.63 |  | 0.83 (0.52-1.34) | 0.15 |
| *Case-control* | 3 | 2.32 (0.92-5.83) | 0.07 |  | 2.10 (0.76-5.82) |  |
| Region | 40 |  | 0.002 | 20.2 |  | 0.007 |
| *East Asia* | 21 | Ref. |  |  | Ref. |  |
| *Southeast Asia* | 1 | 2.50 (0.75-8.39) | 0.13 |  | 1.31 (0.24-7.16) | 0.75 |
| *West Asia* | 1 | 16.34 (4.95-53.94) | <0.0001 |  | 17.80 (5.00-63.32) | <0.0001 |
| *East and southeast Asia* | 1 | 4.52 (1.37-14.89) | 0.02 |  | 3.98 (1.10-14.43) | 0.04 |
| *North America* | 10 | 1.23 (0.79-1.92) | 0.35 |  | 1.14 (0.62-2.10) | 0.66 |
| *Australia* | 1 | 1.30 (0.40-4.27) | 0.65 |  | 1.43 (0.38-5.47) | 0.59 |
| *Europe* | 5 | 2.09 (1.17-3.74) | 0.01 |  | 1.75 (0.89-3.43) | 0.10 |
| Mean age | 40 | 1.38 (0.86-2.21) | 0.18 | 2.31 | 0.97 (0.57-1.65) | 0.91 |
| Male, % | 40 | 0.20 (0.36-1.09) | 0.06 | 6.48 | 0.43 (0.07-2.49) | 0.33 |
| Publication, year | 40 | 1.02 (0.70-1.50) | 0.92 | 2.63 | 1.05 (0.73-1.53) | 0.77 |
| Quality grade | 40 | 0.80 (0.59-1.08) | 0.14 | 3.12 | 1.03 (0.76-1.40) | 0.82 |

CI, confidence interval; MAFLD, metabolic associated fatty liver disease; OR, odd ratio.

**Table S11. Characteristics of the included studies in the incidence density of type 2 diabetes among NAFLD patients**

| Author | Country | Study year | Publication year | Male (%) | Average age | Period | Sample size | Population of NAFLD | Cases | Person-year | Diagnosis of NAFLD | Quality grade |
| --- | --- | --- | --- | --- | --- | --- | --- | --- | --- | --- | --- | --- |
| Xiaoping Tang *et al* | China | 2006-2016 | 2022 | 72.7 | 62.65 | 11 | 5,326 | 696 | 86 | 7,656 | Hepatic ultrasonography | 5 |
| Xintian Cai *et al* | Japan | 2004-2015 | 2022 | 82.6 | 44.38 | 5.1 | 1,820 | 1,820 | 167 | 9,282 | Hepatic ultrasonography | 7 |
| Wen Dai *et al* | China | 2011-2014 | 2021 | 45.8 | 51 | 5 | 12,649 | 1,216 | 177 | 6,080 | Hepatic ultrasonography | 8 |
| Yejin Kim *et al* | Korea | 2011-2018 | 2022 | 53.2 | 37.28 | 5.3 | 245,054 | 72,537 | 8,381 | 326,013 | Hepatic ultrasonography | 8 |
| Haojie Zhang *et al* | UK | 2006-2010 | 2022 | 44.2 | 57 | 11 | 365,339 | 90,748 | 8,774 | 998,228 | Index | 7 |
| Yasuji Arase *et al* | Japan | 1997-2007 | 2009 | 88.3 | 48.8 | 4.9 | 6,003 | 6,003 | 411 | 29,415 | Hepatic ultrasonography | 8 |
| Hyo Jung Cho *et al* | Korea | 2000 | 2019 | 57.6 | 44.2 | 5.18 | 2,726 | 498 | 5 | 2,580 | Mixed diagnostic methods | 7 |
| Tomomi Kogiso *et al* | Japan | 1990-2019 | 2020 | 50.6 | 48 | 7.3 | 544 | 544 | 44 | 3,971 | Liver biopsy | 7 |
| Norio Akuta *et al* | Japan | 2009-2019 | 2018 | 60.9 | 51 | 4.2 | 402 | 402 | 19 | 1,608 | Liver biopsy | 6 |
| Goh Eun Chung *et al* | Korea | 2009-2012 | 2021 | 56.1 | 30.7 | 8.6 | 5,254,786 | 488,510 | 55,150 | 4,201,186 | Index | 7 |
| Arun J. Sanyal *et al* | USA | .. | 2021 | 36.0 | 52 | 4 | 1,773 | 1,026 | 206 | 4,256 | Liver biopsy | 6 |
| Zihan Wei *et al* | China | 2007-2014 | 2019 | 50.6 | 69.4 | 8 | 5,561 | 2,508 | 552 | 20,064 | Hepatic ultrasonography | 7 |
| Wei-Dong Li *et al* | China | 2008-2012 | 2015 | 66.5 | 52.7 | 3.85 | 4,847 | 1,412 | 245 | 5,436 | Hepatic ultrasonography | 7 |
| Kristina Önnerhag *et al* | Sweden | 1978-2006 | 2019 | 70.2 | 52.5 | 19.5 | 120 | 105 | 61 | 2,048 | Liver biopsy | 7 |
| Cheng Yu *et al* | China | 2011-2013 | 2022 | 63.5 | 45.23 | 2.28 | 23,713 | 1,986 | 192 | 4,528 | Hepatic ultrasonography | 7 |
| Chan Liu *et al* | China | 1994-2016 | 2022 | 54.5 | 44.8 | 1.06 | 15,464 | 2741 | 223 | 16,446 | Hepatic ultrasonography | 7 |
| Takuya Fukuda *et al* | Japan | .. | 2016 | 63.6 | 41.5 | 12.8 | 4,629 | 730 | 176 | 9,344 | Hepatic ultrasonography | 7 |
| Patrik Nasr *et al* | Sweden | 1988-1993 | 2020 | 69.0 | 49.3 | 17.5 | 106 | 106 | 66 | 1,855 | Liver biopsy | 7 |
| Yang Xia *et al* | China | 2013-2019 | 2021 | 51.6 | 43.73 | 3.69 | 24,602 | 6,705 | 565 | 24,736 | Hepatic ultrasonography | 8 |
| Chang-Hoon Lee *et al* | Korea | 2009-2013 | 2021 | 67.0 | 45.1 | 5.1 | 3,003,068 | 623,018 | 86966 | 3,177,392 | Index | 7 |
| Ji Cheol Bae *et al* | China | 2005-2009 | 2018 | 68.9 | 44.8 | 3.95 | 7,849 | 3,668 | 308 | 14,350 | Hepatic ultrasonography | 7 |
| LeonA.Adams *et al* | Australia | 1994-2005 | 2009 | 68.4 | 52.9 | 11.1 | 358 | 106 | 20 | 1,177 | Index | 8 |
| Yoshiharu Tokita *et al* | Japan | 2004-2013 | 2017 | 64.1 | 45.4 | 10 | 2,408 | 270 | 39 | 2,700 | Hepatic ultrasonography | 6 |
| Xiaodan Zheng *et al* | Japan | 2004-2015 | 2021 | 52.1 | 44.78 | 6.05 | 14,280 | 2,515 | 324 | 15,216 | Hepatic ultrasonography | 7 |
| V.W.-S.Wong *et al* | China | 1996-2003 | 2004 | 52.4 | 47 | 3.5 | 42 | 42 | 11 | 147 | Liver biopsy | 8 |
| Hossein Bahrami *et al* | Iran | 1999-2001 | 2003 | 60.4 | 37.8 | 2 | 53 | 53 | 3 | 106 | Mixed diagnostic methods | 6 |
| Vincent J. H. Yao *et al* | USA | .. | 2021 | 52.9 | 56 | 5 | 4,609 | 652 | 230 | 3,260 | Index | 7 |
| Jian-Gao Fan *et al* | China | 1995-2002 | 2006 | 90.5 | 39.31 | 6 | 1,146 | 358 | 62 | 2,148 | Hepatic ultrasonography | 7 |
| Yuya Seko *et al* | Japan | 2013-2019 | 2019 | 48.0 | 57 | 3 | 173 | 173 | 73 | 519 | Liver biopsy | 8 |
| I. Friis-Liby *et al* | Sweden | 1994-2001 | 2004 | 77.0 | 46 | 2.8 | 102 | 80 | 6 | 224 | Mixed diagnostic methods | 8 |
| A. Y. Hui *et al* | China | .. | 2005 | 65.0 | 41.8 | 6.1 | 21 | 17 | 3 | 104 | Liver biopsy | 8 |
| Jiwoo Lee *et al* | Korea | .. | 2019 | 73.6 | 50.49 | 4.3 | 6,240 | 2,830 | 344 | 15,154 | Hepatic ultrasonography | 7 |
| Alfred Sidney Barritt *et al* | USA | 2004-2007 | 2011 | 70.0 | 57.9 | 3.75 | 118 | 21 | 13 | 78 | Liver biopsy | 7 |
| Yusuke Kawamura *et al* | Japan | 1997-2010 | 2011 | .. | 49 | 5.6 | 6,508 | 6,508 | 536 | 36,445 | Hepatic ultrasonography | 8 |
| Dong Hyun Sinn *et al* | Korea | 2003-2013 | 2019 | 52.1 | 48.7 | 4 | 51,463 | 15,842 | 5370 | 63,368 | Hepatic ultrasonography | 8 |
| Jie Ming *et al* | China | 2007-2013 | 2015 | 42.3 | 47.8 | 5 | 508 | 97 | 10 | 485 | Hepatic ultrasonography | 7 |

NAFLD, non-alcoholic fatty liver disease.

**Table S12. Characteristics of studies reporting the incidence density of type 2 diabetes in patients with NAFLD: source of heterogeneity**

|  | Studies, n | Median | Mean | Range |
| --- | --- | --- | --- | --- |
| Mean age, years | 36 | 47.9 | 48.3 | 30.7-69.4 |
| Male, % | 35 | 60.9 | 61.2 | 36.0-90.5 |
| Follow up period, year | 36 | 5.05 | 6.3 | 1.1-19.5 |
| Publication year | 36 | 2019 | 2017 | 2003-2022 |
|  | Studies, n | Total person year of type 2 diabetes  (inter-study range) | | Range  (Incidence density, per 1,000-person year) |
| Diagnosis of NAFLD |  |  | |  |
| *Index* | 5 | 998.228 (3,260-31,773,921) | | 17.0 (8.8-70.5) |
| *Hepatic ultrasonography* | 19 | 14,350 (5,758-22,400) | | 21.3 (11.2-84.7) |
| *Liver biopsy* | 9 | 1,608 (147-2,047.5) | | 35.6 (11.1-165.1) |
| *Mixed ^a^* | 3 | 224 (165-1,401.8) | | 26.8 (1.9-28.3) |
| Region |  |  | |  |
| *East Asia* | 27 | 9,282 (2,639.8-22,400) | | 21.3 (1.9-140.7) |
| *West Asia* | 1 | .. | | .. |
| *North America* | 3 | 3,260 (1,669.4-3,758) | | 70.6 (48.4-165.1) |
| *Australia* | 1 | .. | | .. |
| *Europe* | 4 | 1,951.3 (1,447.3-251,092.6) | | 28.3 (8.8-35.6) |

NAFLD, non-alcoholic fatty liver disease.

^a^ Mixed diagnostic methods refer to the definition of NAFLD in a study using more than one diagnostic method.

**Table S13. Average annual percent change (%) in the incidence density of type 2 diabetes among NAFLD populations globally and by region**

|  | Years reported | AAPC (95% CI) | P value |
| --- | --- | --- | --- |
| Global | 1978-2013 | 6.9 (-62.0 to 200.6) | 0.90 |
| By region |  |  |  |
| East Asia | 1990-2013 | 21.5 (0.5 to 46.9) | <0.001 |
| West Asia | 1999 | .. | .. |
| North America | 2004 | .. | .. |
| Australia | 1994 | .. | .. |
| Europe | 1978-2006 | -5.3 (-9.9 to -0.5) | <0.001 |

AAPC, average annual percent change, CI, confidence interval; NAFLD, non-alcoholic fatty liver disease.

**Table S14. The incidence density of type 2 diabetes among patients with NAFLD-stratified by age, region, publication year, sample size and diagnosis of NAFLD (excluding index and hospital record of NAFLD)**

|  | Studies, n | Incidence density (95% CI) | *I^2^* |
| --- | --- | --- | --- |
| Global | 31 | 25.3 (20.1-31.8) | 100.0% |
| Average age, years old |  |  |  |
| *<47.8* | 15 | 20.4 (17.1-24.3) | 99.9% |
| *≥47.8* | 16 | 30.7 (20.0-47.0) | 100.0% |
| Region |  |  |  |
| *East Asia* | 25 | 22.2 (17.1-28.7) | 100.0% |
| *West Asia* | 1 | 28.3 (23.5-34.0) | - |
| *North America* | 2 | 90.8 (25.7-274.1) | 99.3% |
| *Europe* | 3 | 30.8 (26.5-35.7) | 95.1% |
| Publication year |  |  |  |
| *<2019* | 14 | 26.8 (22.2-32.2) | 99.9% |
| *≥2019* | 17 | 23.9 (16.7-34.2) | 100.0% |
| Quality grade |  |  |  |
| *5* | 1 | 11.2 (11.0-11.5) | .. |
| *6* | 4 | 22.1 (9.9-48.6) | 99.9% |
| *7* | 16 | 23.0 (18.0-29.4) | 99.9% |
| *8* | 10 | 33.6 (20.7-54.0) | 100.0% |
| Follow up period, years |  |  |  |
| *<5.0* | 15 | 36.9 (23.9-56.4) | 99.9% |
| *≥5.0* | 16 | 17.7 (14.9-21.0) | 100.0% |
| Diagnosis of NAFLD |  |  |  |
| *Hepatic ultrasonography* | 19 | 22.5 (17.0-29.9) | 100.0% |
| *Liver biopsy* | 9 | 41.9 (24.5-70.6) | 99.9% |
| *Mixed ^a^* | 3 | 11.4 (1.4-87.0) | 99.9% |

CI, confidence interval; NAFLD, non-alcoholic fatty liver disease.

^a^ Mixed diagnostic methods refer to the definition of NAFLD in a study using more than one diagnostic method.

**Table S15. Univariable and multivariable meta-regression analyses on the incidence density of type 2 diabetes among patients with NAFLD**

|  | Studies, n | Univariable analysis | | | Multivariable analysis | |
| --- | --- | --- | --- | --- | --- | --- |
|  |  | OR (95% CI) | *P* value | R^2^ (%) | OR (95% CI) | *P* value |
| Region | 36 | 1.45(1.02-2.05) | 0.04 | 9.23 |  | 0.01 |
| *East Asia* | 27 | Ref. |  |  | Ref. |  |
| *West Asia* | 1 | 1.31 (0.25-6.95) | 0.74 |  | 7.82 (1.12-54.56) | 0.04 |
| *North America* | 3 | 4.10 (1.52-11.07) | 0.01 |  | 3.45 (1.24-9.57) | 0.03 |
| *Australia* | 1 | 0.78 (0.15-4.08) | 0.76 |  | 0.59 (0.10-3.56) | 0.55 |
| *Europe* | 4 | 1.09 (0.49-2.41) | 0.83 |  | 1.41 (0.55-3.62) | 0.46 |
| Mean age | 36 | 1.03 (0.99-1.07) | 0.13 | 3.76 | 1.03 (0.99-1.07) | 0.09 |
| Male, % | 35 | 0.98 (0.95-1.01) | 0.17 | 2.73 | 0.98 (0.95-1.01) | 0.13 |
| Publication, year | 36 | 0.98 (0.93-1.02) | 0.30 | 0.34 | 0.97 (0.92-1.02) | 0.21 |
| Diagnosis | 36 | 1.05 (0.80-1.39) | 0.72 | 2.57 |  | 0.22 |
| *Hepatic ultrasonography* | 19 | Ref. |  |  | Ref. |  |
| *Index* | 5 | 0.92 (0.41-2.09) | 0.84 |  | 0.84 (0.36-1.97) | 0.68 |
| *Liver biopsy* | 9 | 1.90 (0.98-3.66) | 0.06 |  | 1.35 (0.69-2.62) | 0.37 |
| *Mixed ^a^* | 3 | 0.50 (0.18-1.37) | 0.17 |  | 0.20 (0.06-0.70) | 0.01 |
| Quality grade | 36 | 1.30 (0.88-1.94) | 0.19 | 2.35 | 1.46 (0.97-2.19) | 0.07 |
| Follow-up years | 36 | 0.96 (0.89-1.03) | 0.23 | 1.33 | 0.98 (0.92-1.05) | 0.53 |

CI, confidence interval; NAFLD, non-alcoholic fatty liver disease; OR, odd ratio.

^a^ Mixed diagnostic methods refer to the definition of NAFLD in a study using more than one diagnostic method.

**Table S16. Characteristics of the include studies in the incidence density of type 2 diabetes among MAFLD patients**

| Author | Country | Study year | Publication year | Male (%) | Average age | Sample size | Population of MAFLD | Events | Period | Person-year | Quality grade |
| --- | --- | --- | --- | --- | --- | --- | --- | --- | --- | --- | --- |
| Cheng Yu *et al* | China | 2011-2013 | 2022 | 63.5 | 45.23 | 23,713 | 2,191 | 231 | 2.28 | 4,995 | 7 |
| Teruki Miyake *et al* | Japan | 2003-2017 | 2022 | 47.7 | 40 | 9,459 | 1,881 | 139 | 10 | 18,810 | 6 |
| Yu-Ming Cheng *et al* | Taiwan, China | 2008-2022 | 2022 | 42.6 | 54.92 | 9,719 | 1,150 | 277 | 14 | 16,100 | 7 |

MAFLD, metabolic associated fatty liver disease.

**Table S17. Characteristics of studies reporting the incidence density of type 2 diabetes in patients with MAFLD: source of heterogeneity**

|  | Studies, n | Median | Mean | Range |
| --- | --- | --- | --- | --- |
| Mean age, years | 3 | 45.2 | 46.7 | 40.0-54.9 |
| Male, % | 3 | 47.7 | 51.3 | 42.6-63.5 |
| Publication year | 3 | 2022 | 2022 | 2022 |
|  | Studies, n | Total patients of type 2 diabetes  (inter-study range) | | Incidence density  (inter-study range, per 1,000-person year) |
| Region |  |  | |  |
| *East Asia* | 3 | 16,100 (10,547.7-17,455) | | 17.2 (12.3-31.7) |

MAFLD, metabolic associated fatty liver disease.

**Table S18. The incidence density of type 2 diabetes among patients with MAFLD**

|  | Studies, n | Incidence density (95% CI) | I2 |
| --- | --- | --- | --- |
| China | 3 | 26.9 (7.3-44.4) | 100.0% |
| Quality grade |  |  |  |
| *6* | 1 | 7.4 (7.3-7.5) | 100.0% |
| *7* | 2 | 28.3 (10.6-73.2) | 100.0% |

CI, confidence interval; MAFLD, metabolic associated fatty liver disease.
